# Role of Soil Biofilms in Clogging and Fate of Pharmaceuticals: A Laboratory-Scale Column Experiment

**DOI:** 10.1021/acs.est.3c02034

**Published:** 2023-08-09

**Authors:** Edinsson Muñoz-Vega, Stephan Schulz, Paula Rodriguez-Escales, Vera Behle, Lucas Spada, Alexander L. Vogel, Xavier Sanchez-Vila, Christoph Schüth

**Affiliations:** † Institute of Applied Geosciences, Technische Universität Darmstadt, Darmstadt 64287, Germany; ‡ Department of Civil and Environmental Engineering, Universitat Politècnica de Catalunya, Barcelona 08034, Spain; § Hydrogeology Group (UPC−CSIC), Barcelona 08034, Spain; ⊥ Institute for Atmospheric and Environmental Sciences, Goethe-University Frankfurt, Frankfurt am Main 60438, Germany; ∥ Water Resources Management Division, IWW Water Centre, Mülheim an der Ruhr 45476, Germany

**Keywords:** biofilms, clogging, soil organic matter, redox potential, pharmaceuticals, double porosity model

## Abstract

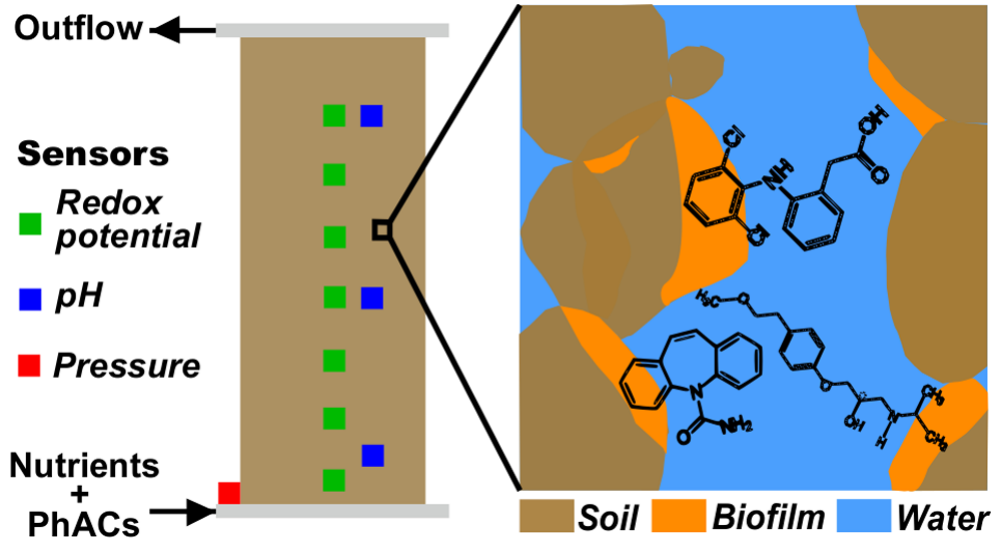

Contamination of groundwater with pharmaceutical active compounds (PhACs) increased over the last decades. Potential pathways of PhACs to groundwater include techniques such as irrigation, managed aquifer recharge, or bank filtration as well as natural processes such as losing streams of PhACs-loaded source waters. Usually, these systems are characterized by redox-active zones, where microorganisms grow and become immobilized by the formation of biofilms, structures that colonize the pore space and decrease the infiltration capacities, a phenomenon known as bioclogging. The goal of this work is to gain a deeper understanding of the influence of soil biofilms on hydraulic conductivity reduction and the fate of PhACs in the subsurface. For this purpose, we selected three PhACs with different physicochemical properties (carbamazepine, diclofenac, and metoprolol) and performed batch and column experiments using a natural soil, as it is and with the organic matter removed, under different biological conditions. We observed enhanced sorption and biodegradation for all PhACs in the system with higher biological activity. Bioclogging was more prevalent in the absence of organic matter. Our results differ from works using artificial porous media and thus reveal the importance of utilizing natural soils with organic matter in studies designed to assess the role of soil biofilms in bioclogging and the fate of PhACs in soils.

## Abstract 2

This study reports biosorption for pharmaceuticals of different speciation and a reduction of clogging in the presence of soil organic matter.

## 1 Introduction

The presence of pharmaceutical active compounds (PhACs) in the aquatic environment is ubiquitous^1,2^ and is related to anthropogenic activities, such as disposal of effluents from wastewater treatment plants (WWTPs) into water bodies.^3^ In the case of groundwater, in addition to natural infiltration of PhACs-loaded surface waters, irrigation with reclaimed water^4^ and techniques such as soil aquifer treatment (SAT), river bank filtration (RBF), and managed aquifer recharge (MAR), coupled to WWTP effluents, might constitute sources for PhACs in the subsurface.^5,6^

Once in aquifers, the most important mechanisms for natural attenuation of PhACs are biotransformation and sorption.^7,8^ Both processes are of complex nature, because they are influenced by PhACs physicochemical properties (e.g., presence of functional groups, polarity) as well as by hydrogeochemical and hydraulic conditions of the soil-water matrix (e.g, pH, redox potential (*Eh*), organic matter (OM) content, and hydraulic conductivity (*K*)), challenging the study of their interrelationship and requiring interdisciplinary approaches.^9,10^ Of special relevance is the pH of the environment because it determines the ionic form of PhACs (defined by p*K*_a_) and the charges on the mineral and organic surfaces of the soil.

In redox active zones, which are usually present in SAT, RBF, and MAR systems, microbial biomass is organized and immobilized by the formation of biofilms.^11^ Biofilms are composed of active biomass, extracellular polymeric substances (EPS), and soluble microbial products (SMP).^12^ EPS are self-produced insoluble gel-type structures, embedding the active cells.^13^ It comprises mostly polysaccharides, proteins, and humic and uronic acids. SMP, sometimes also referred to as soluble EPS,^14,15^ are defined as soluble cellular components released during substrate metabolism and cell lysis.^16^ PhACs biotransformation occurs mainly in the biofilms since they concentrate most of the biological activity. Furthermore, biofilms can affect the physical characteristics of the hosting porous medium by increasing the internal cohesion of soil microaggregates^17^ and by colonizing the pore space, resulting in a reduction of hydraulic conductivity, a phenomenon known as bioclogging.^18^ Nonbiological mechanisms for clogging include accumulation of particulate matter and precipitation of minerals due to chemical processes.^19^ The relevance of each clogging mechanism varies from case to case but, in general, bioclogging has been pointed out as one of the most important processes reducing *K*.^19−21^

When biofilms start to develop in soils, they occupy a fraction of the available porosity.^22^ This phenomenon can be conceptualized by dividing the original porosity (η) into two domains, one occupied by the biofilm (immobile porosity, η_im_) and the other one available for flowing water (mobile porosity, η_m_).^23^ To account for mobile and immobile porosity at the Darcy scale, the porous media can be handled accordingly with a dual domain approach. The two separate domains coexist at the same location and exchange mass at a rate proportional to the difference in concentrations,^24^ resulting in a non Fickian transport behavior.^25^ Previously, this approach has been referred to as a two region model^24^ or, equivalently, dual porosity model.^23,25^

Organic pollutants diffuse into and out of biofilms, being the predominant solute transport process within cell clusters.^26^ Diffusion is driven by disequilibrium between the concentration in the portion of porosity that allows water flow and that occupied by biofilm. The growth of biofilm might also result in the retention of water in the form of immobile porosity,^22,25^ which can retain, at the same time, PhACs. In addition, due to the presence of diverse functional groups in the biofilm structure, mainly in the EPS,^11^ and to the fact that PhACs first must reach and cross the biofilm layer to contact the supporting substrate,^27^ it is expected that biofilms influence the sorption behavior of PhACs.^28^

The literature regarding sorption of PhACs onto biofilms is scarce, partly contradictory and it is mainly focused in biosolids of wastewater sludges^29,30^ and biomass in moving bed biofilm reactors (MBBRs).^31−33^ In the case of natural soils, there are very few studies available. Bertelkamp et al.^28^ found negligible sorption of PhACs onto soil biomass comparing breakthrough curves of biological active and inactive soil columns. Similar results were reported elsewhere.^34,35^ On the other hand, Rodriguez-Escales and Sanchez-Vila^6^ postulated that soil biomass is a potentially relevant sorbent for UV filters of anionic speciation, validating their assumption with geochemical modeling of biotic and abiotic batch experiments with aquifer sediments, conducted by Liu et al.^36^

As the influence of soil biofilms on the fate of PhACs in the subsurface remains not sufficiently understood, further investigations are required to elucidate the contribution of these biological structures to processes such as flow and solute transport including sorption and biotransformation of PhACs. To investigate this, we performed column experiments using diluted synthetic wastewater and a natural soil under biotic and abiotic conditions, as well as with the organic matter removed from the soil. We hypothesize that due to the different soil surfaces and environments, both redox conditions and biofilm growth will be different, and therefore, hydraulic conditions related to bioclogging, as well as sorption and biotransformation of PhACs will vary. We hereby aim to contribute to a better characterization of the influence of soil biofilms on clogging and the fate of PhACs in soils.

## 2 Role of Biofilm on the Fate of PhACs: Theoretical Background

### 2.1 Conservative Processes: Advection, Diffusion (dual domain), and Dispersion

Conservative transport processes in the two region model used in this study are described by two equations. For the mobile region, advection and dispersion are included in the transport equation, which is extended by a term that describes the diffusive exchange with the immobile region, controlled by a first order rate ω (eq 1). For the immobile region, neither advection nor dispersion are relevant and only the diffusive exchange with the mobile region is considered (eq 2).^24^
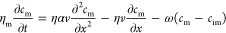
1
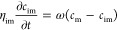
2where *c*_m_ and *c*_im_ [ML^–3^] are the concentrations of solutes in the mobile and immobile liquid region, respectively. α [L] is the longitudinal dispersivity. *v* [LT^–1^] is the pore-water velocity. ω [T^–1^] represents a first order mass transfer coefficient between the mobile and immobile regions. The ratio between η_m_ and η is defined as β [-].

### 2.2 Sorption

Sorption is a general term that includes a number of processes where a dissolved compound interacts with the solid phase (sorbent) and is retained in a reversible way. The portion of sorbed mass in a given space and time is related to the concentration in the liquid phase by a partition coefficient, , with *c*_*s*_^*i*^ [MM^–1^] and *c*_*l*_^*i*^ [ML^–3^] being the equilibrium concentrations of the *i* compound, in the solid and liquid phases, respectively. The latter expression is also referred to as linear isotherm.^37^

Usually, sorption of neutral compounds is related to their retention by organic phases in the soil, such as organic carbon^6,7^ and additionally, but less commonly, by biofilms.^38^ Li et al.^39^ recently presented new models to predict the *K*_d_ of PhACs, incorporating soil and molecular parameters, based on multilinear and nonlinear regression models of batch experiments. The expression for neutral PhACs is^39^
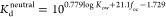
3where *K*_ow_ represents the octanol–water distribution coefficient, which is constant for neutral PhACs. *f*_oc_ is the fraction of organic carbon in the soil.

When PhACs are in their ionic form, ionic interactions could be relevant^40^ and strongly dependent on the chemical conditions of the flowing water (pH), the actual PhAC (defined by p*K*_a_), and the charge of the solid surface (characterized by the pH and the point of zero charge, PZC). Following this, Li et al.^39^ also reformulated the expressions of sorption for ionic forms of PhACs:
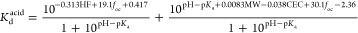
4
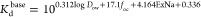
5where *D*_ow_ represents the pH-dependent octanol–water distribution coefficient. CEC (cmol/kg) and ExNa (cmol/kg) are the cation exchange capacity and exchangeable sodium, respectively. HF [-] is the hydrophilic factor (Section S1), and MW [g/mol] is the molecular weight of the PhAC.

Commonly sorption isotherms are not linear, and therefore the Freundlich isotherm is used. To avoid ambiguities of units, a solubility-normalized version of the Freundlich isotherm was previously proposed:^41,42^
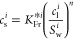
6where *S*_w_^*i*^ [ML^–3^] is the water solubility of the compound. *K*_Fr_^*i^ [MM^–1^] is the unit equivalent Freundlich coefficient. *n* is the Freundlich exponent. For linear isotherms (*n* = 1) eq 7 results.
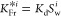
7

### 2.3 Degradation

Degradation of PhACs involves breaking up or transformation of the molecule. This process is usually mediated, either directly or indirectly, by microorganisms. A first-order degradation model assumes the degradation rate μ [T^–1^] being proportional to the concentration of the degraded compound, i.e. . In the dual-domain model, degradation could take place in both regions (mobile and immobile) as well as in the liquid and solid phase. Assuming μ being everywhere the same, as suggested by Van Genuchten and Wagenet^24^ to decrease the number of fitting parameters, we obtain^43^
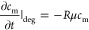
8
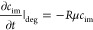
9where *R* [-] is the retardation factor, , with ρ_b_ [ML^–3^] the bulk density of the media.

### 2.4 Integrated Model

The model used in this work incorporates all of the processes described above. Based on the two region model proposed by Van Genuchten and Wagenet,^24^ it describes the transport of solutes in an aggregated medium, where the advective–dispersive flux is constrained to a mobile liquid region that exchanges solutes with an immobile zone by diffusion. Sorption, described by linear isotherms, and degradation can occur in both regions:
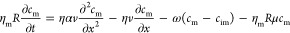
10
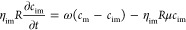
11

## 3 Materials and Methods

### 3.1 PhACs and Soil

The three PhACs used in this study were Carbamazepine (CBZ), Diclofenac (DIC), and Metoprolol (MET) (Merck, Germany). Their structure and chemical properties are listed in Table S1. A stock solution of the three PhACs was prepared in a 2:1 mixture of acetonitrile and methanol with a concentration of 500 mg L^–1^ of each PhAC, and then stored at 4 °C.

A loamy sand, collected from the B Horizon of a grassland (coordinates: 49°49′36′′N, 8°32′31′′E) in the alluvial plain of the Hessian Ried, Germany, was used for the experiments. The selection of soil was based on several studies showing considerable contamination of PhACs in topsoils where irrigation^4,44−46^ or infiltration^47−49^ with treated/untreated wastewater occurred. In addition, other authors have shown the vulnerability of the upper soil layers in areas impacted with WWTPs effluents,^50^ such as the Hessian Ried,^51,52^ where the studied soil was derived.

The selected soil was dried for 6 h at 105 °C to minimize the biological activity of the native bacterial community^53^ and sieved through a 2 mm mesh. A subsample of the soil was then heated in a muffle furnace at 550 °C for 8 h to remove the organic matter, which is referred hereafter as muffled soil (MS), as in other studies.^54^ Physicochemical properties and mineralogical composition of the original organic soil (OS) and the MS are presented in Table 1 and Section S2.

**Table 1:** Physical and Chemical Properties of Both Soils

Parameter	Organic soil (OS)	Muffled soil (MS)
Mean particle size [mm]	0.15	0.18
Organic matter content [%]^a^	3.1	0.0
Organic carbon content [%]^b^	0.6	0.0
pH^c^	8.0	9.5
CEC [cmol/kg]^d^	9.6	4.7
ExNa [cmol/kg]^d^	0.07	0.18

a Loss on ignition.

b TOC analyzer.

c In 0.01 M CaCl_2_, according to ISO 10390:2020.^55^

d Determined according to ISO 11260:2018.^56^

### 3.2 Experiments

We performed two sets of experiments. The first one was based on abiotic batch experiments aimed at characterizing the sorption properties of the OS and MS with respect to the three selected PhACs. The experiments were performed according to the respective OECD guideline.^57^ Details of the batch experiments can be found in Section S3.

**Figure 1:**
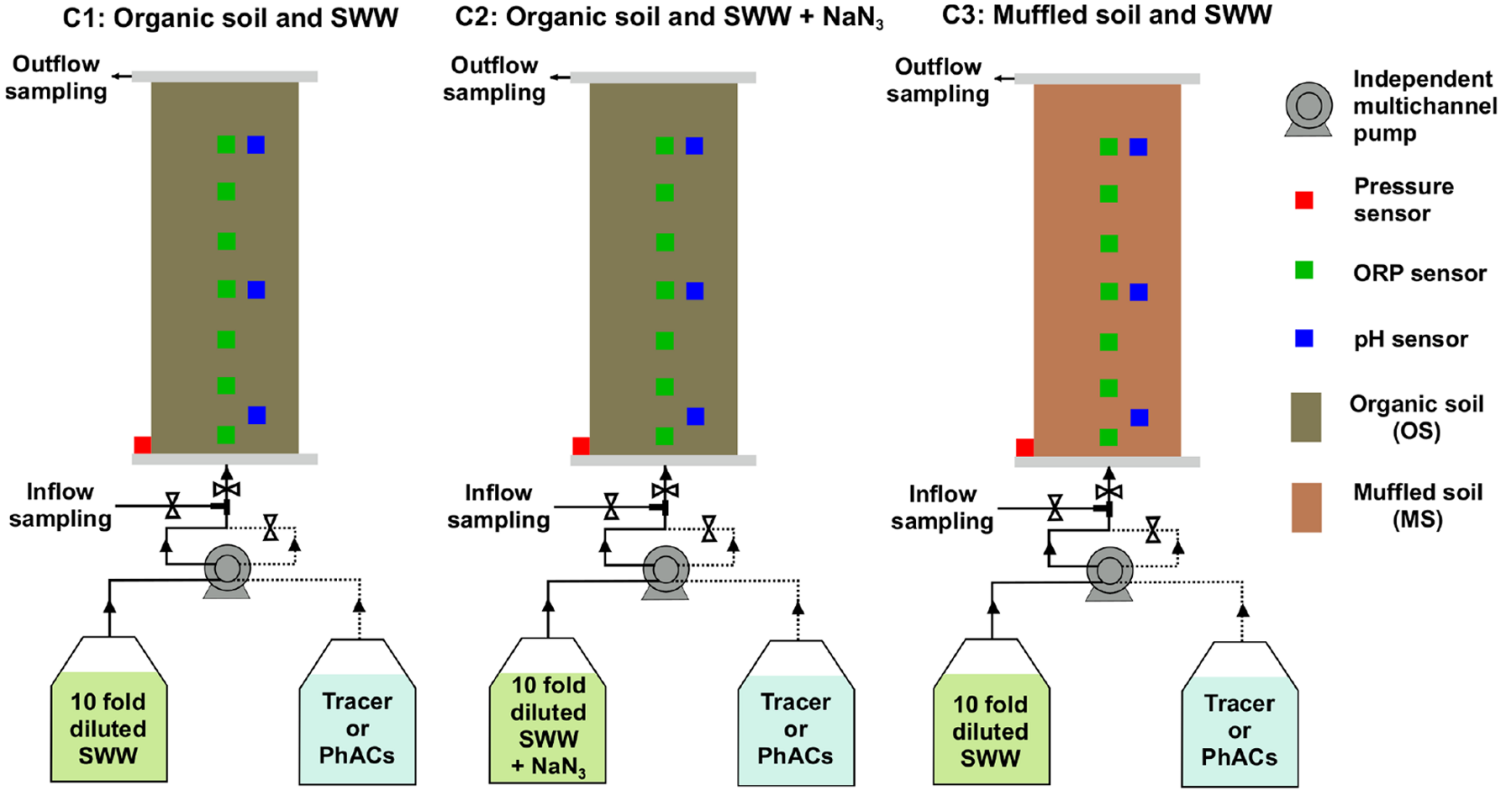
Scheme of column experiments.

Second, column experiments were performed using three acrylic glass columns (11 cm i.d., 40 cm length), gradually packed with the soil in 1 cm increments under saturated conditions. Columns 1 (C1) and 2 (C2) were filled with OS and column 3 (C3) with MS (Figure 1). The columns were designed for long experimental times and with high spatial and temporal monitoring resolution for physical, chemical, and biological parameters. Consequently, single individual columns were used for the three different experimental approaches.

To prevent the leaching of fine particles, a porous glass plate of 8 mm thickness (ROBU, Hattert, Germany) and pore diameters between 16 and 40 μm was placed at the bottom and top of all columns. The columns were operated in the upflow direction under saturated conditions and fed by two peristaltic pumps (Ismatec, Switzerland) equipped with short PharMed tubing (2.54 mm i.d., Coleparmer, Germany) and then connected to the bottom of the columns by stainless steel tubing (1.52 mm i.d.). A second feeding line was established, joined to the principal tubing with a 3-way connector and a system of valves, allowing inline mixing of the feeding solution with tracer or PhACs solutions, when required (Figure 1). The feeding solution consisted of synthetic wastewater (SWW), also prepared according to the respective OECD recipe^58^ and diluted 10-fold with PhACs-free tap water (section S4). For C2, 1 g of sodium azide (NaN_3_) per liter was added to the diluted SWW to inhibit biological activity, similar to other column studies addressing biotransformation of PhACs.^28,59,60^ All columns were first operated for several pore volumes (PVs) with diluted SWW but without PhACs, aiming for conditioning the biomass (approximately 200 PVs for C1 and 150 for C2 and C3). Afterward, during a second stage, which lasted about 50 PVs, the feeding solution was mixed inline (Figure 1) in a 5:1 ratio with a solution containing 0.006% v/v of the stock solution (i.e., 30 μg L^–1^ of each of the three PhACs), which was prepared weekly in ultrapure water, resulting in a nominal concentration of 5 μg L^–1^ of each PhAC entering to all columns. In the third stage, again, only diluted SWW was injected until the end of the experiment. During the full duration of the experiment, the columns were continuously operated with a flow rate of 864 mL day^–1^ (seepage velocity = 9.1 cmd^–1^), readjusted when required, corresponding to average water residence times of about 2 days, which was estimated based on the total porosity fitted with tracer experiments (Table 2) to account for the exchange with the immobile phase.

**Table 2:** Fitted Parameters of the Two-Region Model for the Three Tracer Experiments Conducted for Each Column^a^

Parameter	C1			C2			C3		
PV [-]	10	70	end	10	71	end	10	75	end
β [-]	0.455 ± 0.002	0.462 ± 0.001	0.606 ± 0.003	0.900 ± 0.006	0.996 ± 0.004	1.000 ± 0.000	0.907 ± 0.005	0.774 ± 0.005	0.805 ± 0.002
η [-]	0.443 ± 0.001	0.430 ± 0.001	0.377 ± 0.001	0.501 ± 0.003	0.499 ± 0.002	0.472 ± 0.003	0.431 ± 0.001	0.468 ± 0.001	0.444 ± 0.001
η_m_ [-]	0.202 ± 0.001	0.198 ± 0.001	0.228 ± 0.001	0.451 ± 0.004	0.498 ± 0.002	0.472 ± 0.003	0.391 ± 0.002	0.362 ± 0.003	0.357 ± 0.001
η_im_ [-]	0.241 ± 0.002	0.231 ± 0.001	0.148 ± 0.001	0.050 ± 0.005	0.002 ± 0.003	0.000 ± 0.004	0.040 ± 0.002	0.106 ± 0.003	0.087 ± 0.001
ω [day^–1^]	0.204 ± 0.003	0.190 ± 0.001	0.319 ± 0.006	0.023 ± 0.004	0.023 ± 0.023	0.046 ± 0.044	0.148 ± 0.015	0.788 ± 0.030	0.661 ± 0.012
α [cm]	0.767 ± 0.045	0.613 ± 0.022	0.231 ± 0.022	2.221 ± 0.088	1.645 ± 0.046	3.200 ± 0.083	0.294 ± 0.014	0.057 ± 0.008	0.087 ± 0.004

a Values are reported with the 95% confidence interval calculated by CXTFIT.

*Eh* along the column lengths was monitored continuously by in situ oxidation reduction potential (ORP) probes (Paleo Terra, Netherlands), which were vertically placed inside of each column, in conjunction with a AgCl (3 M KCl) reference electrode. Every probe contained seven equidistant (5 cm) platinum sensors, from 2 to 32 cm from the inflow section (Figures 1 and S1). ORP values were adjusted to *Eh* adding 212 mV.^61^ Furthermore, three soil pH probes (ecoTech, Germany) were horizontally installed at 3, 17, and 32 cm from the inflow zone of each column along with a 3 M KCl reference electrode. *Eh* and pH data were recorded using data loggers (CR800 and CR1000X, Campbell Scientific, USA). Concentrations of dissolved organic carbon (DOC) and O_2_ as well as major ions (NO_3_^–^, NO_2_^–^, NH_4_^+^, SO_4_^2–^, Ca^2+^, Mn^2+^, and Fe^2+^) were monitored periodically at the inflow and outflow of each column during the whole duration of the experiment. DOC was measured with a carbon analyzer (LiquiTOC II, Elementar, Germany) and major ions with an ion chromatograph (Metrohm 882 Compact IC plus, Germany). Iron was analyzed by ICP-MS (Analytik Jena Plasma Quant MS, Germany).

To measure the changes in hydraulic conductivity (*K*), pressure transducers (SICK, Germany) were installed in the inflow section of each column. Pressure data were also recorded with a data logger (CR1000X, Campbell Scientific). Since the flow rate was known, the pressure difference between inflow and outflow as a function of time was converted to *K* values using Darcy’s law, similar to other studies.^23,62^ To quantify the influence of clogging on porosity and dispersion, three tracer experiments were conducted for each column at 10 and 75 pore volumes (PVs) as well as at the end of the experiment. Sodium bromide (NaBr) was used as tracer for C1 and C3 and tap water in the case of C2, due to the potential interference with the high concentration of NaN_3_. Electrical conductivity was recorded continuously at the outflow during the tracer tests with a conductivity probe (TetraCon 925, WTW, Germany) placed in a flow-through cell. Conservative tracers as well as PhAC breakthrough curves were fitted to eqs 10 and 11 using the code CXTFIT.^43^

PhACs determination and biofilm quantification are described in S6 and S7, respectively.

## 4 Results and Discussion

### 4.1 Hydrochemical Conditions: Evolution of *Eh* and pH

**Figure 2:**
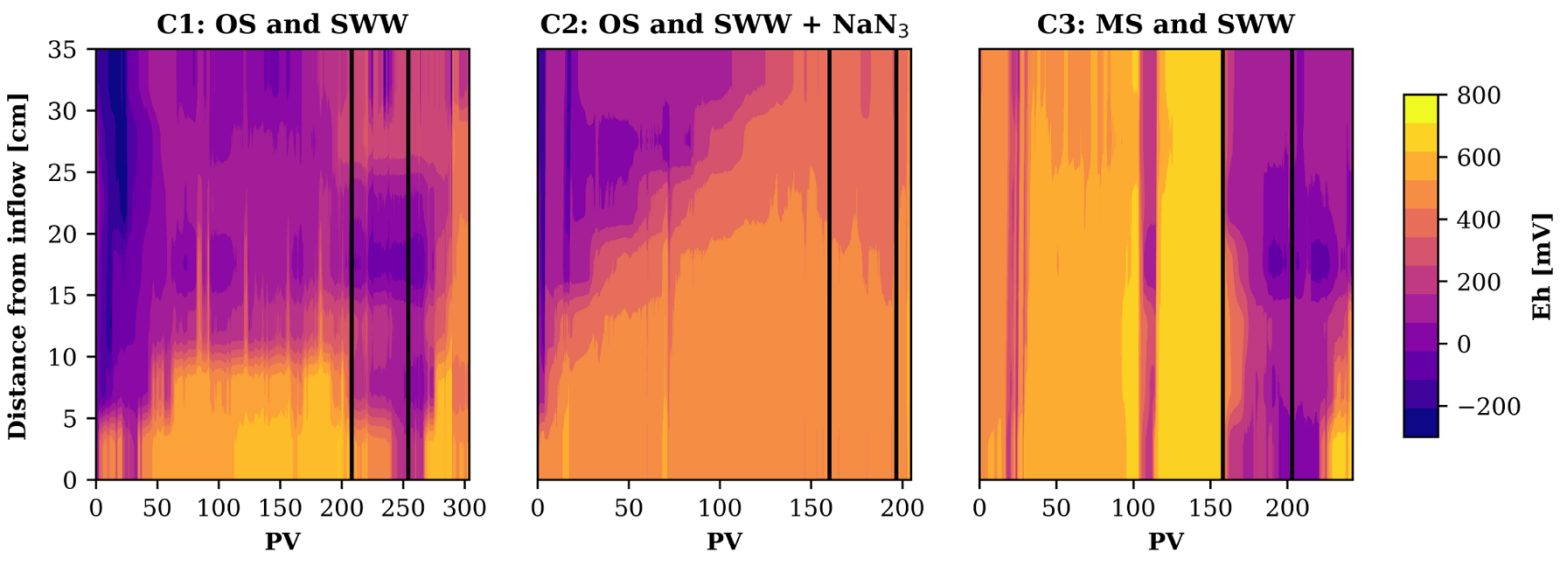
Spatiotemporal evolution of *Eh* in the three columns. Black lines mark the beginning and the end of the PhACs injection.

Both sorption and biotransformation of PhACs are highly influenced by hydrochemical conditions, and we therefore monitored *Eh* and pH *in situ* as well as the evolution of hydrochemistry at the outflow of the columns (Figure 2 and Figures S2 to S10). *Eh* is an indicator of the presence of active biomass and the degree of oxidation of DOC.^63^ The three columns showed very different patterns in the spatiotemporal evolution of the redox levels (Figure 2). C1, the column with original soil and fed with diluted SWW, was in general the one that showed the most reducing conditions. It was characterized by a small oxic zone (*Eh* > 600 mV) close to the inflow (5 to 10 cm), which then declined sharply along the flow path to an anoxic-reduced zone (*Eh* < 100 mV), indicating a very intense consumption of electron acceptors. This assumption was supported by the absence of dissolved oxygen (DO) at the outflow, the transformation of NO_3_^–^, and the mobilization of Mn^2+^ and Fe^2+^ (Figures S3, S4, S9, and S10). These more reducing conditions were associated with higher DOC levels, apparently from the release of soil organic matter (SOM).

In the case of C2, the addition of NaN_3_ to the feeding solution resulted in higher *Eh* values (100–400 mV) within the column. In line with this, DO levels in the outflow samples were at suboxic (defined by DO < 0.0625 mM^64^) and anoxic conditions (Figure S3) and again, mobilization of Mn^2+^ and Fe^2+^ was observed (Figures S9 and S10) despite non denitrification conditions (Figure S4), which can be explained by the inhibition of denitrifying bacteria by NaN_3_.^65,66^ Thus, the behavior of C2 was similar to that of C1, but with less biological activity imposed by the presence of NaN_3_. Apparently, even the quite high concentration of NaN_3_ (1 g L^–1^) was not capable to completely inhibit microbial activity, which has already been reported in previous studies.^59^ However, there was a clear trend of increasing *Eh* values as a function of time over the entire column, indicating a decrease in biological activity over time.

C3, the column without OM, was the only one depicting predominantly oxic conditions before the injection of PhACs, suggesting a low activity of biomass, associated with aerobic respiration. Consequently, neither denitrification nor Mn and Fe mobilization occurred, and high DO concentrations were observed in the outflow samples (Figures S3, S4, S9, and S10). While there was little variation in the *Eh* values over depth, there was a slight variation in time toward increasing *Eh* values prior to the injection of PhACs.

Based on the observations from the three columns, we conclude that differences in redox processes between the three systems depend on the presence of organic matter in the soil, which determines the availability of organic carbon as an electron donor and thus the presence of active microorganisms. During the first 150 PVs, DOC concentrations in the outflow were higher than in the inflow of C1 and C2, indicating DOC release from SOM (Figure S2). Also, DOC concentrations were higher in C2 than in C1, which is consistent with the lower microbial activity induced by NaN_3_. Interestingly, C2 showed more reducing conditions than C3, although C2 was running under microbial inhibition. This can be explained by the fact that C3 was fed with only the organic carbon present in the inflow (around 0.2 mM, Figure S2) and a higher molar concentration of oxygen (between 0.5 and 0.6 mM, Figure S3). Since the molar ratio between oxidation of DOC and the reduction of oxygen is 1:1,^67^ the aerobic conditions of the column are reasonably expectable.

During the injection of PhACs the DOC inflow concentration increased from 0.19 ± 0.05 to 0.26 ± 0.06 mM, which can be attributed to the albeit frugal use of organic solvents (acetonitrile and methanol) for the preparation of the stock solution. Despite this relatively low increase of the inflow DOC concentration (0.0012% v/v of organic solvents in the inflow, significantly lower than in several other studies^61,68−70^), a decrease in *Eh* was observed in all three systems. This effect was particularly pronounced in C3, which changed to anoxic conditions. A possible explanation is the presence of inactive biomass in that column, reactivated by the presence of labile (readily available) DOC, leading to an increase in aerobic respiration in that system. This is in accordance with a greater consumption of electron acceptors (Figures S3 and S4). After the injection of PhACs, i.e., with the return to the feeding solutions of the first stage (diluted SWW without organic solvents), an increase in *Eh* was observed for C1 and C3, demonstrating the effect of the more labile carbon source during the injection of PhACs.

To equilibrate the pH of the soil (OS = 8.0, MS = 9.5) to that of the inflow water, the exchange of 150 PV was required (Figure S11). In C1 and C2, slightly more acidic conditions were observed during the first PVs, which may be related to the production of protons under more aerobic redox conditions.^67^ Simultaneously, calcium concentrations increased (Figure S8), reflecting the possible dissolution of carbonate minerals. During the PhACs injection phase, the pH was constant in depth for every column with values higher than the point of zero charge (PZC, Figure S12). Thus, we assume that the net electric charges of the soils in all columns were negative and constant over depth, despite the different redox zonation.

### 4.2 Effect of Biofilm and SOM in the Hydraulic Evolution of Column Experiments

**Figure 3:**
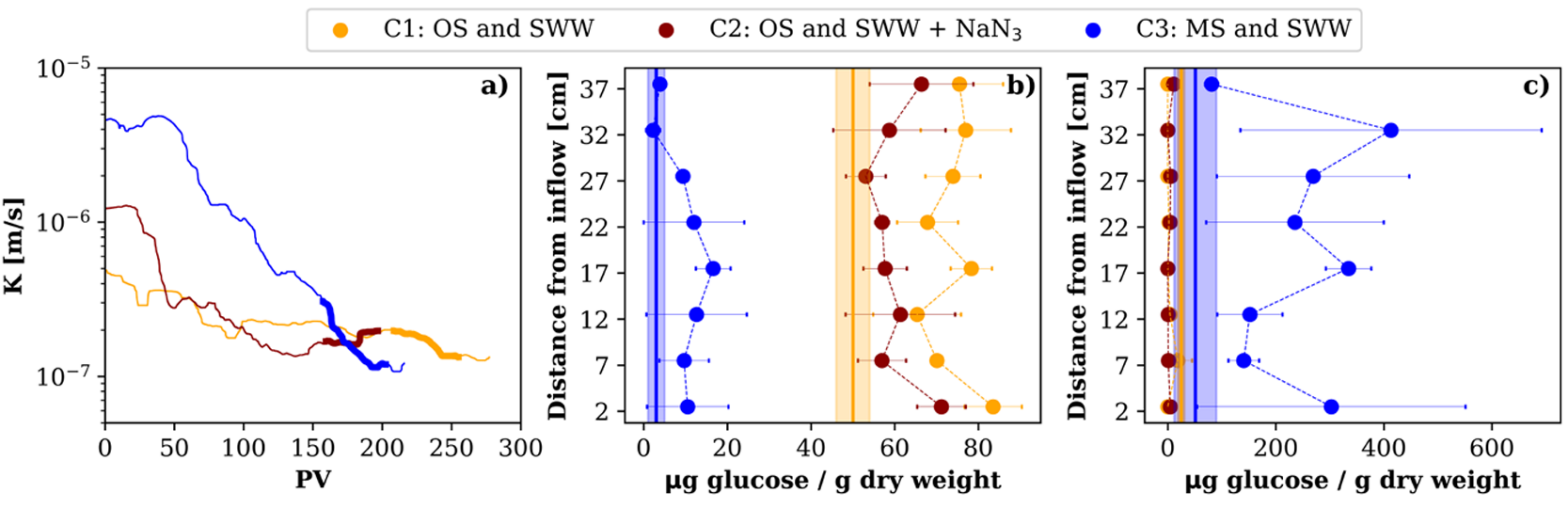
(a) Temporal evolution of the hydraulic conductivity in the three columns. Thicker line represents the period of PhACs injection for each column. A running median filter of 2 PV was applied for noise reduction. Carbohydrates content of the biofilm at the end of the column experiments in (b) EPS and (c) SMP. Error bars represent the standard deviation of triplicate samples. Solid orange and blue lines represent the initial content in the OS and MS, respectively, including standard deviation of triplicate samples (shaded area).

The initial *K* value (*K*_o_) was different in the three columns (C3 > C2 > C1, Figure 3a), which we attribute to the packing. *K* remained approximately constant during the first PVs of the three columns, a period in which C1 and C2 also showed a higher SOM release (Figure S2). However, later in the experiments, all columns experienced a clear decrease of *K* over time (Figures 3a and Figure S13) until the three columns reached a quite similar value (slightly above 10^–7^ m/s).

C3, the column with the lowest biological activity (Section 4.1) and the highest *K*_o_ value, showed the largest reduction of *K*. In contrast, C1, which was the one with the lowest *K*_o_ and highest biological activity, was the column with the least *K* reduction (Figures 3a and S13). This is an apparent contradiction, since clogging is usually driven by the progressive colonization of the porous media by biofilm.^23,25,71^ Nevertheless, the experimental results suggest that SOM plays an important role in the reduction of clogging. It seems that clogging is compensated by a degradation of a portion of the initial SOM (Figure S2), increasing the share of mobile porosity. In the case of C3, due to the absence of SOM, this compensation did not happen and the only mechanism was biological clogging, which is supported by a slight oxidation of DOC by DO (Figures S2 and S3) resulting in *Eh* values depicting subsaturated DO conditions during the first 120 PVs (Figure 2) and also by the fact that DOC accumulation has been explained by biological assimilation in other studies.^72^ In addition, the increase of calcium and the decrease in pH during early PVs in C1 (Figures S8 and S11) indicate dissolution of calcite, which may also compensate clogging.^62^ During the period of PhACs injection, no clear changes in the trend of K decrease were observed despite the evidence of enhanced biological activity, depicted by the decrease in *Eh* values (Figure 2).

The fitted parameters of the dual domain model of the conservative tracers are presented in Table 2 and their observed and modeled breakthrough curves in Figure S14. The first tracer tests (after 10 PVs) are assumed to be representative for the initial conditions, as the evolution of *K* showed no significant changes during the first PVs. All columns initially showed non-Fickian behavior, being more pronounced in C1 (Figure S14). This is reflected in the values of β, equal to 0.46 for C1 and around 0.9 for C2 and C3 (Table 2). This can be explained by a relatively high initial value of η_im_ for C1, which we attribute to the presence of organic matter and an active biofilm (Figure 3b) that acted as a pool for diffusion of the tracer, as noted in other studies.^73,74^

In columns C1 and C2 (both columns with OS), η_im_ tended to diminish with time (i.e., β increases), indicating that SOM was leached or consumed. This was only partially compensated by biofilm production, which should have resulted in an increase in η_im_, as observed in C3 and in other studies.^23,25^ Since C3 contained no SOM, the only process modifying porosity is biofilm growth, indicated by the increase in η_im_. Regarding hydrodynamic dispersion, we would expect an increase in dispersivities due to biocolonization, which has also been reported in other studies.^74,75^ In our case, however, we could not observe such a clear positive trend, and for C1 there even seemed to be a decrease. This unexpected behavior, also noticed by other authors,^23,73,76^ might have been caused by an increase in the mass transfer coefficient between the mobile and immobile phase (ω), revealing enhanced non-Fickianity and thus a higher model complexity, which does not allow for such direct comparisons.

Generally, the parameters derived from the tracer tests indicated non-Fickian transport behavior. The exception was C2, where β = 1 for the second and third tracer tests implies an evolution to Fickian conditions, which can be explained by the presence of NaN_3_ and thus the inhibition of biofilm growth. In this context, it should also be noted that most of the studies describing bioclogging and changes in the transport behavior of porous media have been conducted with glass beads^71^ or quartz sand,^23,25,73,75,77^ substrates neither containing SOM nor variations in surface properties. Our results seem to reveal a complex interaction of processes for natural soils.

To further assess the influence of the biofilm in clogging, we analyzed the different components of the biofilms. Figure 3b and c shows the carbohydrates (CHO) content of the EPS and SMP, which constitutes the dominant component of the biofilms,^16^ extracted from the columns at the beginning and at the end of the experiment. The proteins and humic acid contents are presented in Figure S15. CHO content in EPS increased significantly (*p*-value <0.01) for all columns at all depths, being highest in C1. Additionally, at the end of the experiment we observed significant differences (p-value <0.01) for the EPS-CHO content between all systems, being in the order C1 > C2 > C3. Apparently, the growth of CHO was directly associated with the presence of organic carbon in the columns as well as with the biological activity of the natural bacteria from the soil, since we did not inoculate with microorganisms neither the feed solution nor the soil used.

On the other hand, for SMP-CHO, a significant increase was observed only in C3. The SMP-CHO content in C1 and C2 was practically zero at the beginning as well as at the end of the experiment. This could be explained by the fact that SMP are usually recycled by the microbial community.^15^ Thus, the consumption of SMP-CHO was the lowest in C3, which can be explained by its low biological activity (Figure 2). Another explanation is that the origin of SMP in C3 were from Biomass Associated Products (BAP) rather than Utilization Association Products (UAP), because BAP are associated with environments with less organic carbon.^15^ BAP is more recalcitrant and exhibits lower EPS-protein content, as observed in C3 (Figure S15).

Overall, our work has been able to demonstrate that there is a delicate balance between two processes governing flow and transport. On the one hand, the amount of SOM changes over time, resulting in a significant difference in the space available for biofilm growth, and on the other hand, microorganisms tend to colonize this available space. The net effect is that clogging has the largest impact, in relative terms, in absence of SOM. This finding is in accordance with field results of Nadav et al.,^78^ where heating treatment of an infiltration pond operated with reclaimed water, to reduce the SOM of the facility, did not result in an improvement of the infiltration capacity. Moreover, Valhondo et al.^79^ noticed that the addition of organic layers (compost) in pilot plants for artificial recharge with WWTPs secondary effluent prevented clogging.

### 4.3 Influence of Biofilm on the Fate of PhACs

**Figure 4:**
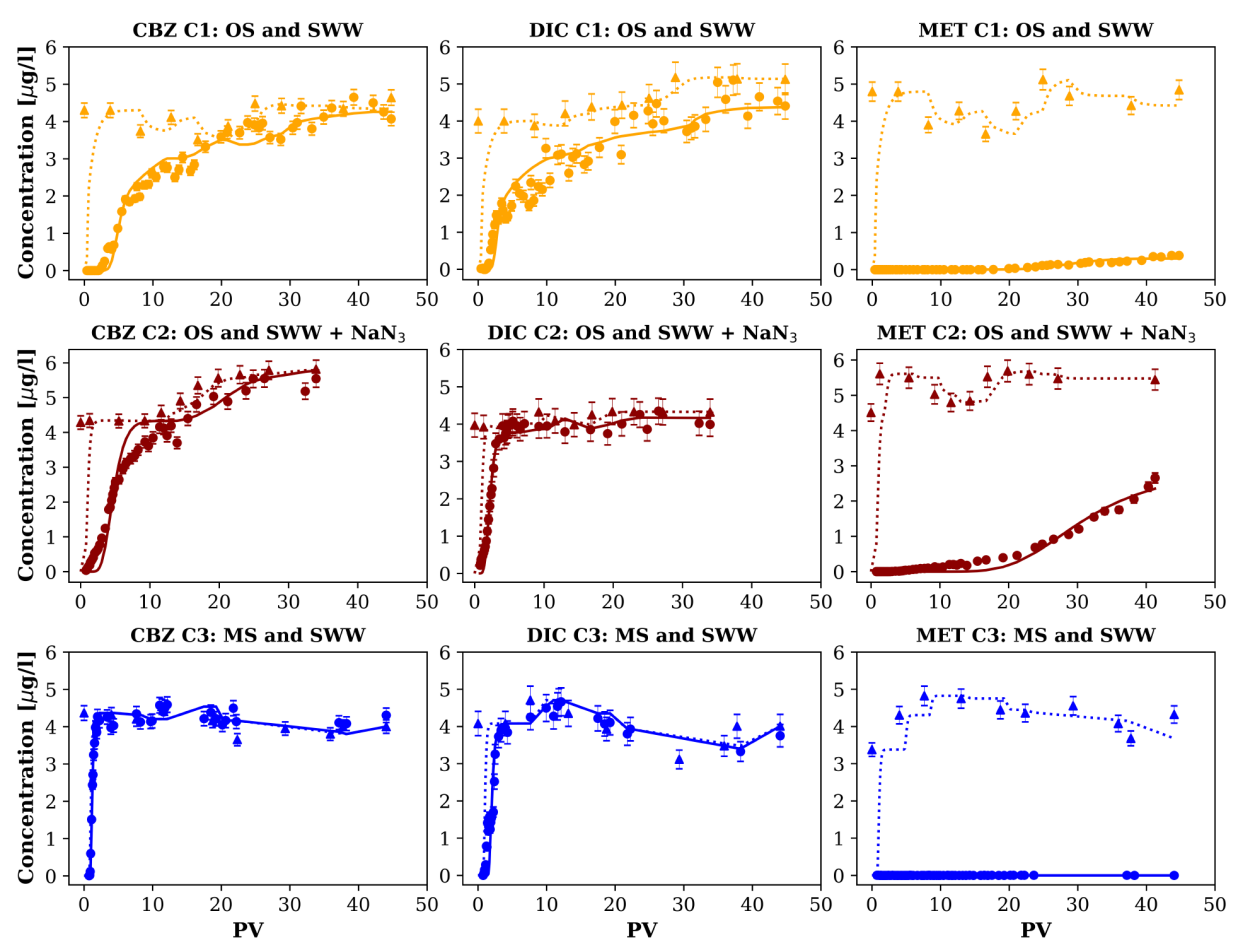
Breakthrough curves of the three compounds and fitted models for each system. Solid and dotted lines represent the integrated and conservative model, respectively. Circles represent outflow concentrations, and triangles inflows. Error bars represent the propagated errors of the solid phase extraction recoveries.

Figure 4 shows the measured and the modeled breakthrough curves (BTCs) of the column experiments considering both sorption and biotransformation (solid line) as well as conservative transport (dashed line) as reference. We used the fitted parameters for both sorption and biotransformation presented in Table S7, and the parameters of the second tracer test for transport (Table 2). For simplicity, both sorption (*R*) and biotransformation (μ) parameters were considered constant along each column.

CBZ fate in the column experiments can be explained by sorption only, confirming its known recalcitrant behavior.^28,60,61^ Its retention was more pronounced in C1 than in C2 and C3, which is reflected by the *R* values (Table S7). Moreover, C1 shows a higher share of the immobile phase (Table 2), which contributed to CBZ retention (Figure 6). Interestingly, CBZ was not retained in batch experiments with muffled sediments (MS, Section S14), meaning that its retention in C3 could be attributed to the existence of a sparse biofilm (Figure 3), which acted as a sorbent. As CBZ was in neutral form at the experimental pH, its retention in the biofilm can be explained by its affinity to more lipophilic compounds present in the biofilm (log *K*_ow_ = 2.45, Table S1). This is similar to the findings of Headley et al.^27^ that suggested lipophilicity as an important factor to explain sorption of neutral pesticides onto river biofilms. Furthermore, Aubertheau et al.^80^ detected CBZ in river biofilms from 12 different sites impacted by WWTPs effluents in the Vienne river, postulating biological uptake as an additional mechanism for sorption of PhACs in biofilms, which could also explain the higher retention of CBZ in C1 compared to C2, due to its higher biological activity (Section 4.1).

Regarding DIC, sorption behavior was similar to that of CBZ, showing higher retention in C1 and lower in C2 and C3. Similarly, whereas retention was observed in C3, batch experiments with the same soil did not show it (Section S14). We also attribute this to the presence of biofilm which probably retained DIC in its more lipophilic portions (log *D*_ow_(pH 8.5) = 0.83, Table S1). Furthermore, as DIC is negatively charged at our experimental pH, it could therefore also sorb electrostatically to positively charged fractions of the EPS, such as amino groups in sugars and proteins, as it was suggested by Rodriguez-Escales and Sanchez-Vila^6^ to describe the enhanced sorption of the UV filter BP-3 in the presence of biofilm. Additionally, DIC has been extracted from river biofilms as well,^80−82^ confirming the possibility of retention of DIC onto biofilms. Besides sorption, DIC was also biodegraded in C1 and C2, but not in C3. These differences in biodegradation can be explained by the different electron acceptors during the PhACs injection. DIC is better degraded under denitrifying and manganese reducing conditions,^83^ both occurring in C1. However, in C2 only manganese reduction could be observed, and in C3 solely denitrification occurred (Figures S4 and S9).

MET behavior was significantly different compared to those of the other compounds, showing high retention in all columns. Sorption was more pronounced in C3, being consistent with batch experiments, which showed more sorption in the muffled soil (Section S14). The different behavior of MET can be explained by its cationic charge at the pH during the experiments, allowing ionic interaction with soil (negatively charged) and biofilm (negatively charged above a pH of 7^82^). Enhanced sorption due to biofilm is a plausible explanation for the higher retention of MET in C1 than that in C2. The electrostatic interaction between cationic PhACs and biofilms was previously observed by Torresi et al.,^32^ where of a total of 23 PhACs studied, only nine having cationic speciation sorbed onto biofilms from moving bed biofilm reactors. MET was also biotransformed in cells C1 and C2. The higher biotransformation in C1 than C2 was attributed to a higher microbial activity and biofilm content (Sections 4.1 and 4.2).

**Figure 5:**
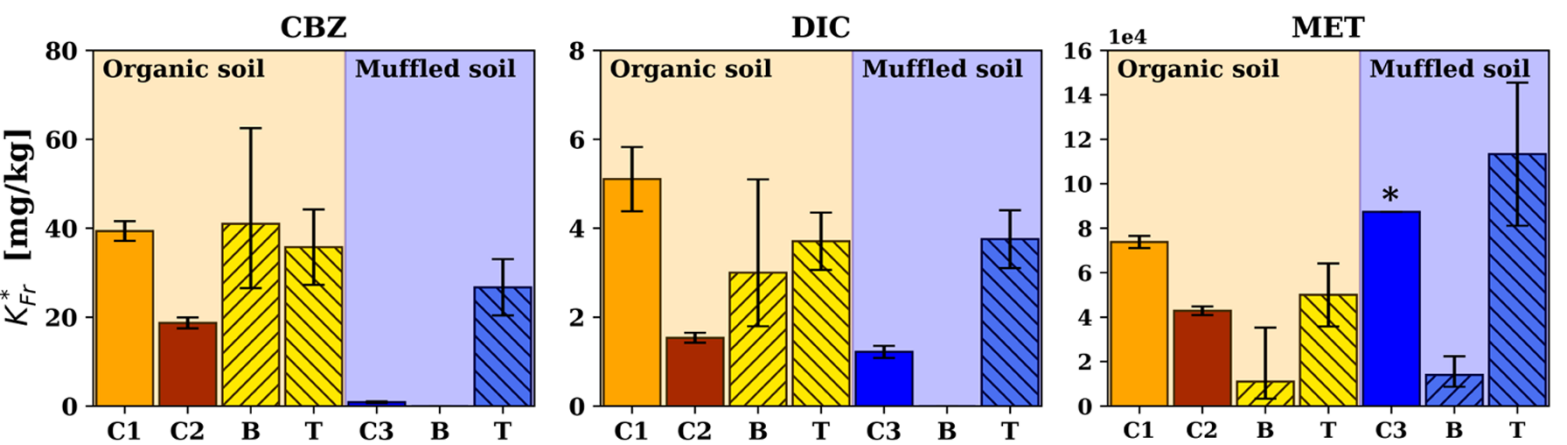
Estimated *K*_Fr_^*^ from column and batch experiments compared to theoretical values. Error bars represent the 95% confidence interval estimated by CXTFIT, the standard deviations of the logarithmic linear regressions, and the standard error of the nonlinear regressions for columns and batch tests as well as theoretical estimations. B = Batch. *T* = theoretical; * represents a minimum value because of no quantification in outflow samples.

The comparison between results of batch and column experiments can be a useful method to explain retardation of organic compounds,^84^ e.g., PhACs,^85^ despite of the inherent differences between the two techniques such as the flow dynamics and the associated contact times between the different phases as well as the soil-water ratio, which have to be considered in the comparison. Figure 5 summarizes *K*_Fr_^*^ for batch experiments, column experiments, and theoretical values estimated using eqs 3–5. *K*_d_ values were converted to *K*_Fr_^*^, as shown in eq 7. Interestingly, in the case of the organic soil, the experimental *K*_Fr_^*^ determined in the column experiments followed the same order of *K*_Fr_^*^ as the theoretical values and the determined ones in the batch experiments. For CBZ and DIC, the observed *K*_Fr_^*^ values in C2 were overestimated by the batch experiments and Li’s model.^39^ We attribute this to the fact that neither Li’s model nor the batch experiments consider the presence of biofilms as an additional sorbent present in the porous media. Besides this, in both C1 and C2 the amount of SOM as a sorbent changed over time because of carbon leaching, which was also not considered in batch experiments and Li’s model. Nevertheless, they reproduced much better the *K*_Fr_^*^ in C1 than in C2, although biofilm growth was more pronounced in C1. This could be due to the fact that the disappearance of SOM as a sorbent was counteracted by the appearance of a new one, i.e., the biofilm. In case of MET the variability of the determined *K*_Fr_^*^ values were higher (from 15000 to 120000 mg/kg). Although the *K*_Fr_^*^ value from Li et al. matched the value observed in C2, the *K*_Fr_^*^ value from the batch experiment underestimated the sorption in the column. This different behavior compared to the other two compounds could be due to the different mechanisms of sorption of MET, which was mainly attributed to ionic retention.

Our results indicate that biological variables in porous media such as the amount and composition of biofilms play a role in the sorption of PhACs of all speciations. This finding differs from the results of Bertelkamp et al.,^28^ one of the few available studies assessing biosorption in soils, where no effect of active biomass was noticed for retention of PhACs, most likely due to the low biological activity in their experiments, expressed by a low DOC removal and the consequently prevalence of oxic conditions, which might be caused by the utilization of a technical sand instead of a soil containing organic phases. Despite that they did not analyze for biofilms in their column experiments, the use of the Fickian advection dispersion model to explain their results allows us to infer that biofilm was not developed under their experimental conditions, conversely to our experiments (Section 4.2).

**Figure 6:**
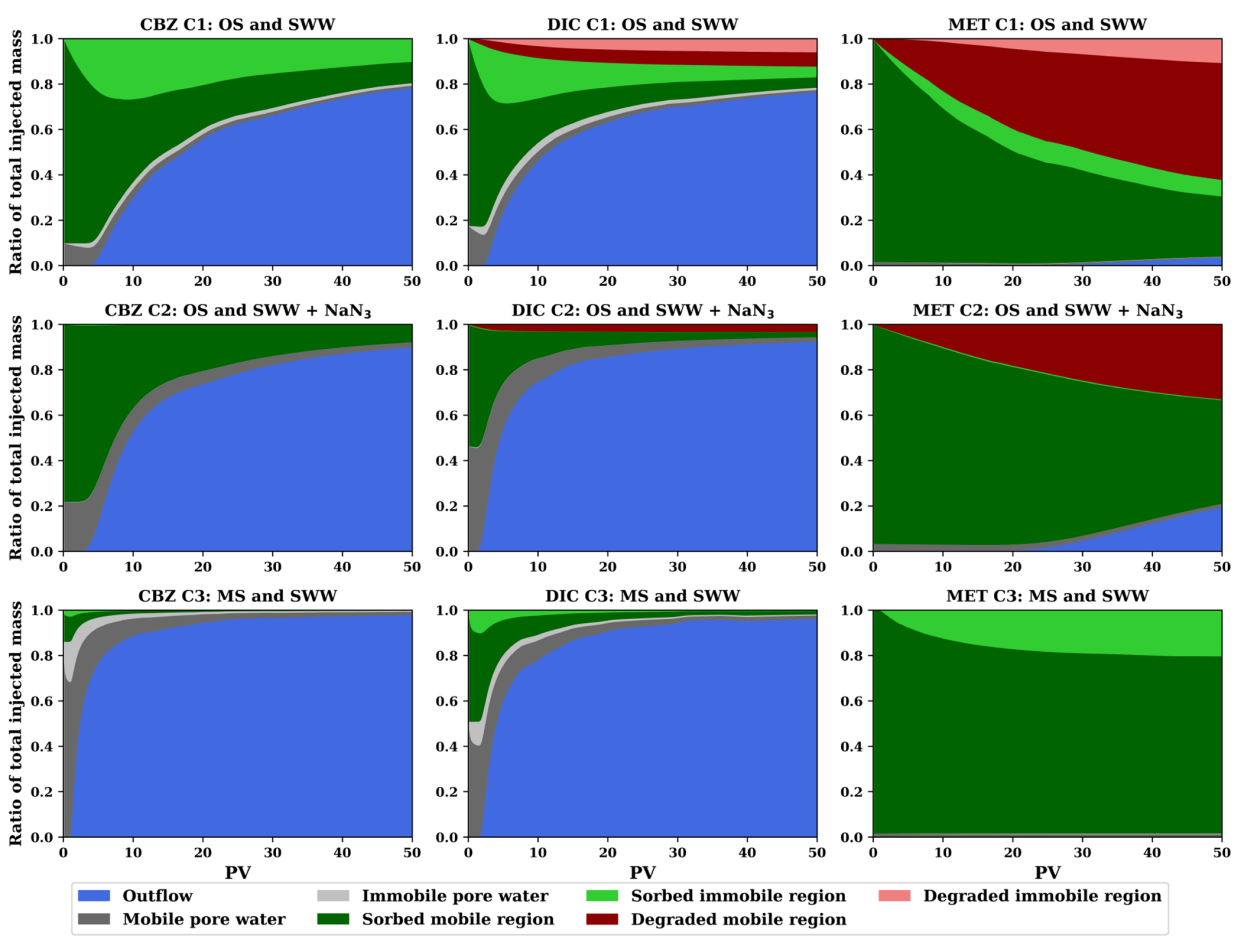
Mass balances of the integrated model for the PhACs in the three columns.

Summarizing the fate of the different PhACs in our study, Figure 6 shows the mass balances along 50 PVs in the different evaluated compartments of the three columns. In C1, the one exhibiting the most non-Fickian behavior caused by the organic matter and biofilm content (Section 4.2), the immobile region comprises an important fraction of the attenuation of all PhACs, in terms of both processes, sorption, and transformation. This is in contrast to the other two columns C2 and C3 with less biological activity (Section 4.1) and lower biofilm content, thus showing more Fickian behavior (Section 4.2).

These findings suggest that the incorporation of natural and biologically active organic soil layers, for instance, in MAR facilities, could result in a better attenuation of PhACs during infiltration, not only increasing biotransformation but also by enhancing their sorption. Likewise, this might even mitigate the typical decrease in infiltration rates observed in field applications. However, it must also be emphasized that our results only allow for initial assumptions, and additional investigations need to be performed in the field to validate our hypotheses. Beside this, future studies could incorporate different types of soils and the use of real treated wastewater to cover a wider range of PhACs and other organic compounds, including their transformation products. Finally, we highlight the importance of multidisciplinary studies aiming to link between biological, chemical, and physical processes in soils, which are of vital importance to preserve groundwater bodies.

## Supporting Information Available

PhACs and soil properties; methods and results of batch experiments; composition SWW; scheme of column experiments; PhACs and biofilm quantification; major ions, pH and normalized hydraulic conductivity evolution of column experiments; observed and modeled tracers; protein and humic acid content of biofilms and fitted parameters of the double porosity model of PhACs (PDF)
