# Supplementary material for: Role of Soil Biofilms in Clogging and Fate of Pharmaceuticals: A Laboratory-Scale Column Experiment
Source: Environ Sci Technol. 2023 Aug 9;57(33):12398–410. doi: 10.1021/acs.est.3c02034 (PMC10448752; doi:10.1021/acs.est.3c02034)
Supplement: Supplementary file 1 — es3c02034_si_001.pdf [file es3c02034_si_001.pdf]

Supporting information for: Role of soil biofilms in clogging and fate of pharmaceuticals: A laboratory-scale column experiment

Edinsson Muñoz-Vega,[*,†] Stephan Schulz,[†] Paula Rodriguez-Escales,[‡,¶] Vera Behle,[‡] Lucas Spada,[§] Alexander L. Vogel,[§] Xavier Sanchez-Vila,[‡,¶] and Christoph Schüth[†,∥]

†*Institute of Applied Geosciences, Technische Universität Darmstadt, 64287 Darmstadt, Germany*

‡*Department of Civil and Environmental Engineering, Universitat Politècnica de Catalunya, 08034 Barcelona, Spain*

¶*Associated Unit: Hydrogeology Group (UPC-CSIC), 08034 Barcelona, Spain*

§*Institute for Atmospheric and Environmental Sciences, Goethe-University Frankfurt, 60438 Frankfurt am Main, Germany*

∥*Water Resources Management Division, IWW Water Centre, 45476 Mülheim an der Ruhr, Germany*

E-mail: edinsson.munoz@tu-darmstadt.de

Contents

S1 PhACs properties **S3**

S2 Soil composition **S3**

S3 Batch experiments **S4**

S4 Composition of feeding solution **S5**

S5 Scheme of the experimental setup for the column experiments. **S6**

S6 PhACs quantification **S7**

S7 Biofilm quantification **S8**

S8 Hydrochemical evolution during the column experiments **S9**

S9 pH evolution of column experiments and determination of PZC **S14**

S10 Temporal evolution of normalized hydraulic conductivity for the column experiments **S15**

S11 Tracer results of column experiments **S16**

S12 Protein and humic acid content of biofilms for column experiments **S17**

S13 Fitted parameters double porosity model for PhACs breakthrough curves in column experiments **S18**

S14 Results batch experiments **S18**

References **S19**

S1 PhACs properties

Table S1: Chemical structure and physicochemical properties of the studied compounds.

Compound	Structure	MW [g/mol]	S_w ^{a} [mg/L]	pK_a	$\log K_{ow}$ ^{a}	$\log D_{ow}$ (pH = 8.5)
CBZ	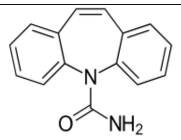	236.27	17.7	13.9[b]	2.45	2.45
DIC	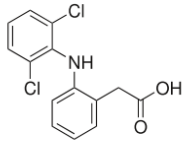	296.15	4.5	4.2[c]	4.51	0.83[d]
MET	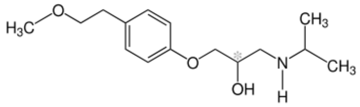	267.37	4777	9.4[c]	1.88	0.73[d]

[a] Estimated with WSKOW v1.42 EPI suite. [b] Jones et al. 2002.[1] [c] Schaffer et al. 2015.[2]
[d] Estimated with ACD/Percepta v14.53.0.

The hydrophilic factor (HF) is a hydrophilicity descriptor calculated based on the number of carbon atoms, the number of hydrophilic groups (-OH, -SH, -NH) and the number of atoms (excluding hydrogen) in a molecule.[3] Values for CBZ, DIC and MET are 0.32, 0.39 and 0.34, respectively.[4]

S2 Soil composition

Table S2: Mineralogical phases of both soils, determined by X-ray diffraction (XRD).

Mineral class	Qualitative	OS [%]	MS [%]
Silicates	Quartz	52	52
	Amphibole	4	4
Feldspar	Albite	19	19
	Alkalifeldspar	12	12
Phyllosilicates	Illite	5	6
	Chlorite	2	2
	Smectite	1	0
Carbonates	Calcite	4	4
	Dolomite	1	1

Table S3: Relative elemental composition of the oxides of both soils, determined by X-ray fluorescence (XRF).

Cation	OS [%]	MS [%]
Si	70.3	73.6
Ti	0.6	0.6
Al	8.2	8.3
Fe	3.9	3.4
Mn	0.2	0.1
Mg	1.6	1.3
Ca	8.3	6.9
Na	3.5	2.9
K	3.3	2.7
P	0.1	0.1

S3 Batch experiments

Batch vessels were prepared by weighing 7.5 g of the respective soil (OS or MS) in 40 mL glass vials with PTFE lined caps. Then, 30 mL of a solution containing 10 mM $CaCl_2$ and 3.08 mM sodium azide (NaN_3) were added to the vessels, the latter to inhibit biological activity. Only abiotic batches were conducted on this study. For preconditioning, batches were shaken at 150 rpm for 12 hours using an orbital shaker (SM 30, Edmund Bühler, Hechingen, Germany). Subsequently, different volumes of the PhACs stock solution were added to each batch to cover six concentration levels ranging from 5 to 1000 μgL^{-1}, always maintaining the volume of organic solvent < 0.1 %. Each level of PhACs concentration was conducted in triplicates for both soils, and control batches, i.e., without soil, were also included. To reach equilibrium conditions, samples were shaken for 4 days, based on equilibration times of 3 days reported for batch experiments utilizing natural sediments with CBZ and DIC,[5] as well as for MET.[6] The batches were then centrifuged at 1900 rpm for 4 hours and supernatant was collected for posterior analyses. The equilibrium sorbed concentrations, C_s [MM^{-1}], were calculated based on the concentration of the control batches:

$$C_s = \frac{V_0(C_0 - C_w)}{m} \tag{S1}$$

where V_0 [L^3] is the volume of the aqueous phase (30 mL), C_0 [ML^{-3}] is the concentration of PhACs in the control batches and m [M] is the mass of soil (7.5 g). C_w [ML^{-3}] is the water equilibrium concentration. Freundlich normalized isotherms were determined with equation 7.

S4 Composition of feeding solution

The feeding solution for column experiments was prepared by the 10-fold dilution of the synthetic wastewater (SWW) with tap water. Final concentrations are presented in Table S4. All compounds were purchased from Merck, Germany. The feeding solution was prepared weekly, and to ensure oxic conditions, air was gently pumped into the inflow reservoirs throughout the experiment using two air pumps (SCHEGO, Germany).

Table S4: Chemical composition of the feeding solution of the column experiments.

Substance	mg/L
Peptone	16.0
Meat extract	11.0
Urea	3.0
K_2HPO_4	2.8
Sodium acetate	2.0
NaCl	0.7
$CaCl_2 \cdot 2H_2O$	0.4
$Mg_2SO_4 \cdot 7H_2O$	0.2

The ionic strength of the feeding solution is 11 mM. In the case of C2, the spiking with sodium azide results in an ionic strength of 22 mM. We consider this difference between both feeding solutions not relevant for the transport of PhACs, based on the results of Bui et al.,[7] where no significant changes in sorption behavior were detected in batch experiments with silica for CBZ and DIC, using ionic strengths of 10 and 20 mM.

S5 Scheme of the experimental setup for the column experiments.

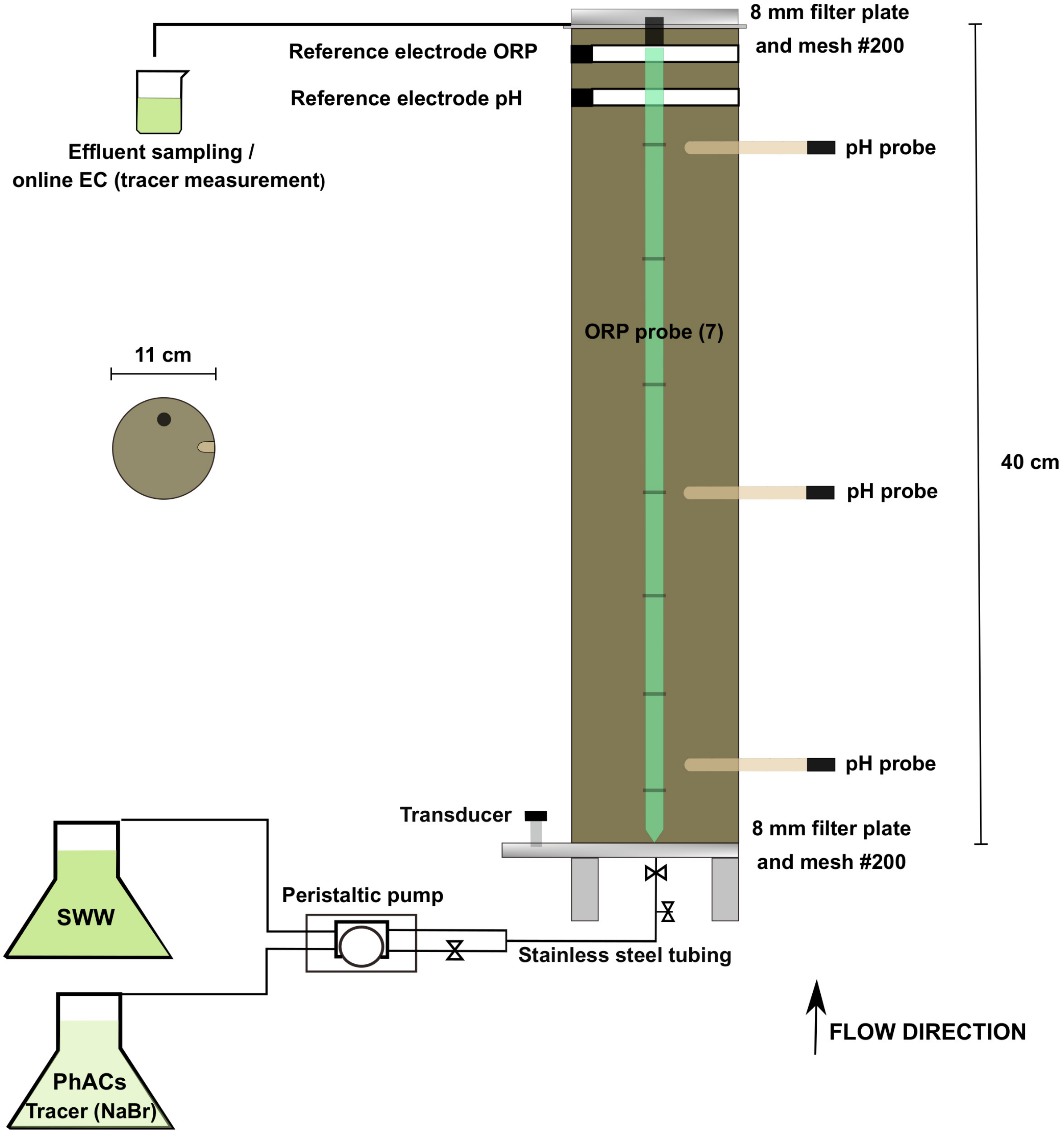

Figure S1: Scheme of column experiments.

S6 PhACs quantification

Batch and column samples for PhACs analyses were fixed to pH = 5 by addition of 0.1 M HCl and stored at 4°C until measurement. Aliquots of 15 mL of PhACs samples plus 85 mL of ultrapure water were enriched by solid phase extraction (SPE) using 3 mL, 60 mg Oasis HLB cartridges (Waters, Germany). Samples were loaded under vacuum in cartridges preconditioned with 1 mL dichloromethane (DCM), 1 mL methanol and 1 mL ultrapure water. The cartridges with the analytes were dried under a stream of N_2 and then eluted under vacuum with 1 mL of a 1:1 DCM/methanol solution. The eluates were collected in 2 mL glass LC-vials and then evaporated with N_2. Afterwards, the residue was dissolved in 200 μL of 10% v/v methanol for posterior quantification of the targeted PhACs (CBZ, DIC and MET).

For quantitative analyses, a volume of 10 μL of sample extract was injected in a UHPLC/HRMS equipped with a heated electrospray ionization (HESI) source. The three PhACs were measured in positive HESI mode. Ultrapure water and acetonitrile, both with 0.1 % formic acid, served as mobile phases. A reversed-phase C18-column (Waters CORTECS T3 2,7 µm 3 mm x 150 mm) was used for the separation of the analytes, with a flow of 0.4 mL min^{-1}. The PhACs concentrations were determined by an internal calibration renewed for every batch of samples. Blanks and quality control standards were included on every set of measurements. Finally, concentration of PhACs were corrected by the SPE recovery rates.

The recovery rates of the SPE were determined by extracting 12 samples of known concentration of the targeted PhACs, six of 5 $\mu g/L$ and six of 10 $\mu g/L$. Mean recovery rates, standard deviations and relative standard deviations (RSDs) are presented in Table S5. Samples of known concentration were added for every batch of SPEs, as additional quality control. Retention times (RT) and limits of quantification (LOQ) of each compound are presented in Table S6. LOQ were calculated based on the standard deviation of the calibration procedure, according to DIN 32645.[8]

Table S5: Recovery rates of the SPE.

Compound	Mean recovery [%]	Standard deviation [%]	RSD [%]
CBZ	92	4	4.3
DIC	79	6	8.0
MET	92	5	5.4

Table S6: RT and LOQ for each substance in the UHPLC/HRMS system.

Compound	RT [min]	LOQ [ng/L]
CBZ	6.62	24
DIC	8.04	38
MET	5.22	42

S7 Biofilm quantification

At the end of the experiment, each column was divided lengthwise into eight sections of 5 cm and biofilm was extracted from three soil samples per homogenized section, following the method of Redmile-Gordon et al.[9] To avoid overestimation of the extra polymeric substances (EPS), soluble microbial products (SMP) were extracted as well. For this, an equivalent of 2.5 g of dry soil was transferred into 50 mL centrifuge tubes and 25 mL of a 0.1 M $CaCl_2$ solution adjusted to pH 7 was added. Then the tubes were shaken at 120 rpm for 30 minutes at 4°C, followed by 30 minutes of centrifugation at 3500 rpm. Subsequently, supernatant, which constitutes the SMP, was collected. To the remaining pellet, 3.85 g of a cation exchange resin (CER Amberlite HPR1100, Merck, Germany) and 25 mL of a phosphate buffer solution (0.76 gL^{-1} $Na_3PO_4 \cdot 12H_2O$; 0.55 gL^{-1} $NaH_2PO_4 \cdot H_2O$; 0.53 gL^{-1} NaCl and 0.08 gL^{-1} KCl) at 4°C and adjusted to pH 7 were added. The pellet was resuspended by hand, shaken at 4°C for 2 hours and finally centrifugated at 4000 rpm for 30 min. The collected supernatant constitutes the EPS of the biofilm. Quantification of proteins and humic acids of the SMP and EPS was performed according to the modified Lowry assay presented by Redmile-Gordon et al.[10] Bovine serum albumin (BSA) and humic acid salt (HA) were used as standards, respectively. Carbohydrates content of the SMP and EPS was measured according to the

Anthrone method,[11] using glucose as standard. To describe the biofilm evolution of column experiments, carbohydrate content of EPS and SMP was measured from triplicates of unused OS and MS. Finally, ANOVA test was used to test for statistically significant differences (p-value < 0.01) in biofilm composition before and after the column experiments for the three systems, using the module scipy 1.7.3 of Python.

S8 Hydrochemical evolution during the column experiments

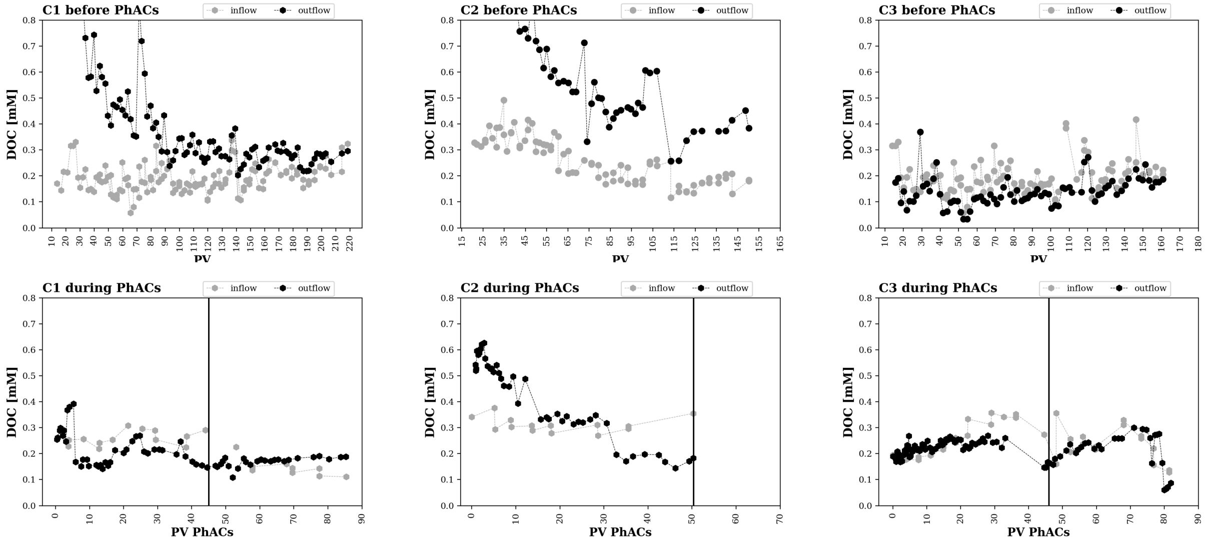

Figure S2: Dissolved organic carbon concentrations before and during the injection of PhACs. Black vertical line shows the end of PhACs injection. The carbon concentrations in ppm were converted to mM, dividing by the molecular weight of carbon, equivalent to 12 g/mol.

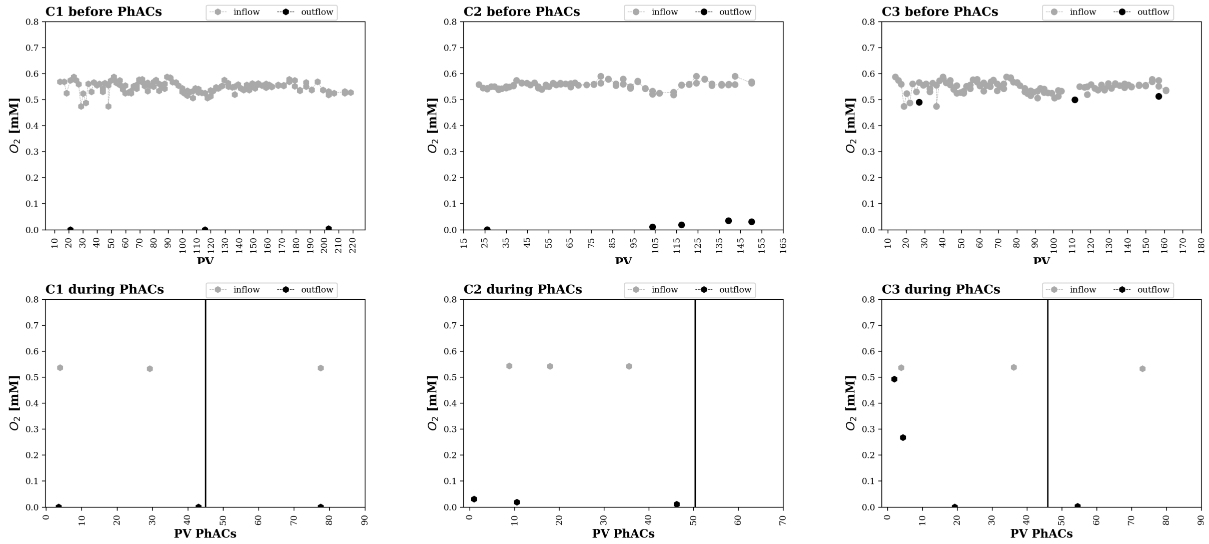

Figure S3: Dissolved oxygen concentrations before and during the injection of PhACs. Black vertical line shows the end of PhACs injection.

To avoid oxygen exchange with the atmosphere, outflow O_2 concentrations were measured with an optical dissolved oxygen sensor (FDO 925, WTW, Germany) placed in a specially designed flow through cell connected to the outflow pipe of each column.

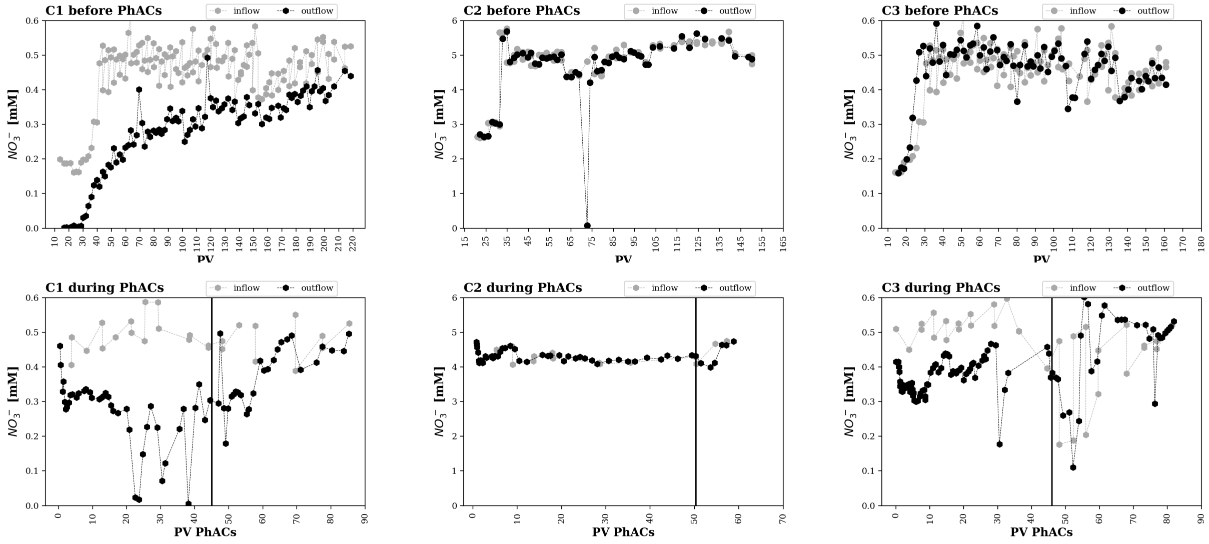

Figure S4: Nitrate concentrations before and during the injection of PhACs. Black vertical line shows the end of PhACs injection.

Nitrate concentrations in C2 were affected by the azide (N_3) concentrations. Despite of this, no reduction in concentrations were observed in the outflow, indicating no denitrifica-

tion.

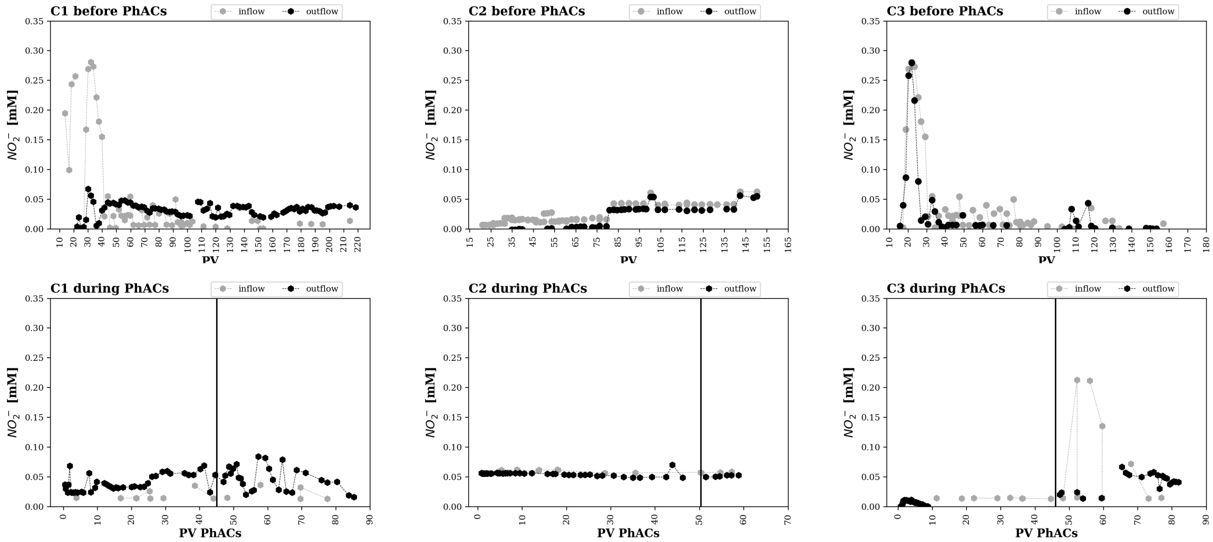

Figure S5: Nitrite concentrations before and during the injection of PhACs. Black vertical line shows the end of PhACs injection.

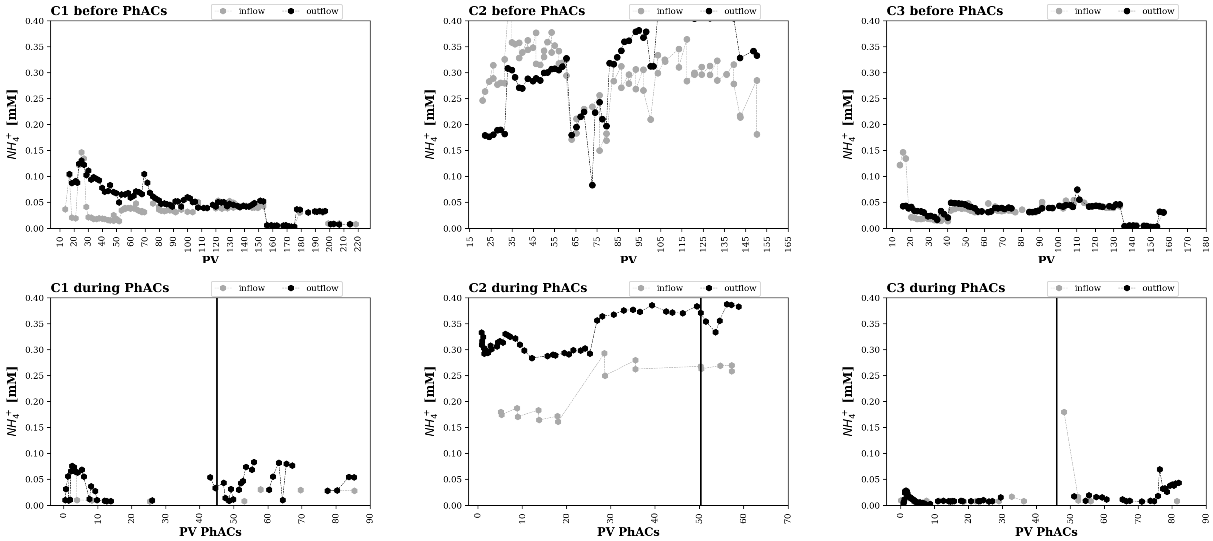

Figure S6: Ammonium concentrations before and during the injection of PhACs. Black vertical line shows the end of PhACs injection.

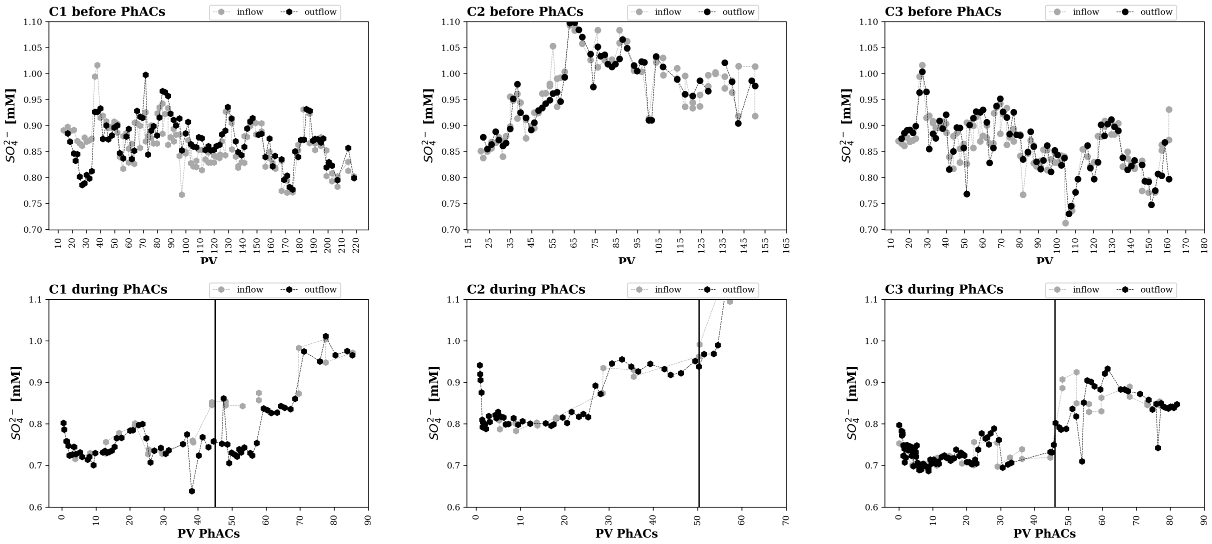

Figure S7: Sulfate concentrations before and during the injection of PhACs. Black vertical line shows the end of PhACs injection.

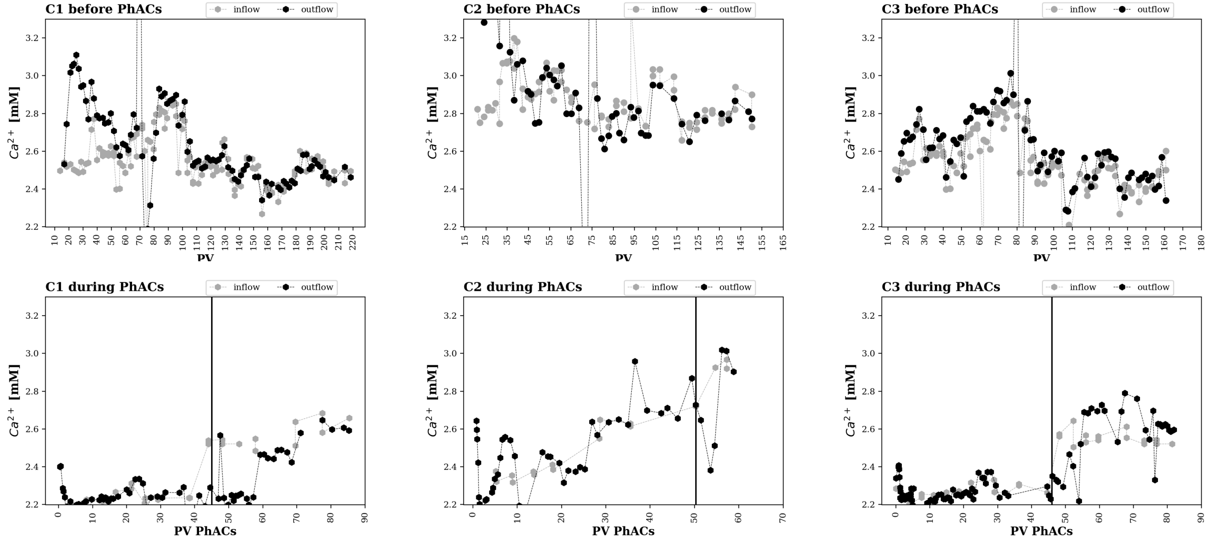

Figure S8: Calcium concentrations before and during the injection of PhACs. Black vertical line shows the end of PhACs injection.

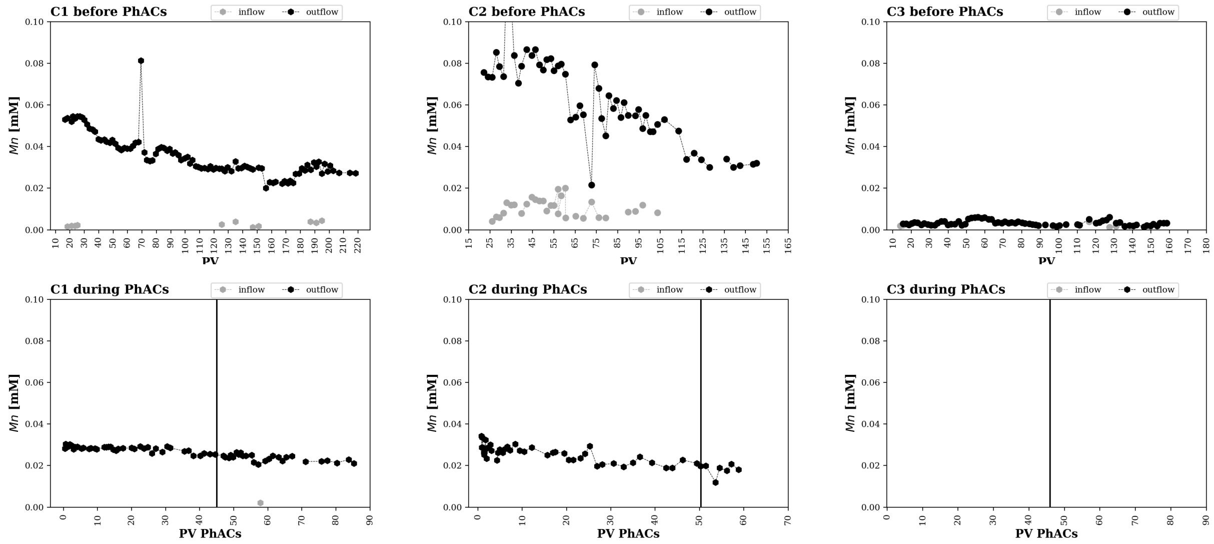

Figure S9: Manganese concentrations before and during the injection of PhACs. Black vertical line shows the end of PhACs injection.

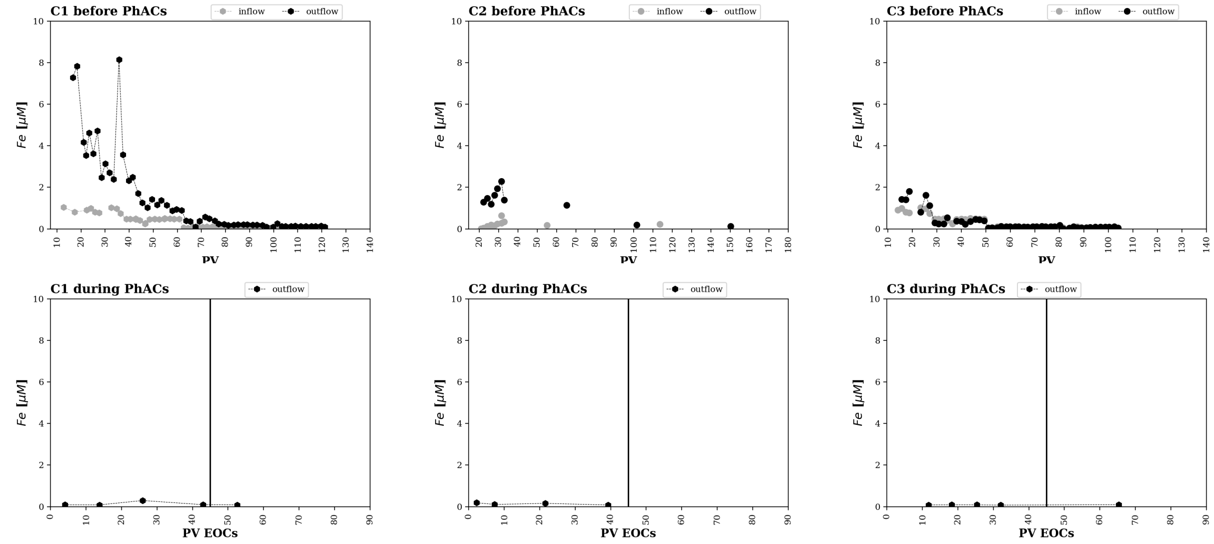

Figure S10: Iron concentrations before and during the injection of PhACs. Black vertical line shows the end of PhACs injection.

S9 pH evolution of column experiments and determination of PZC

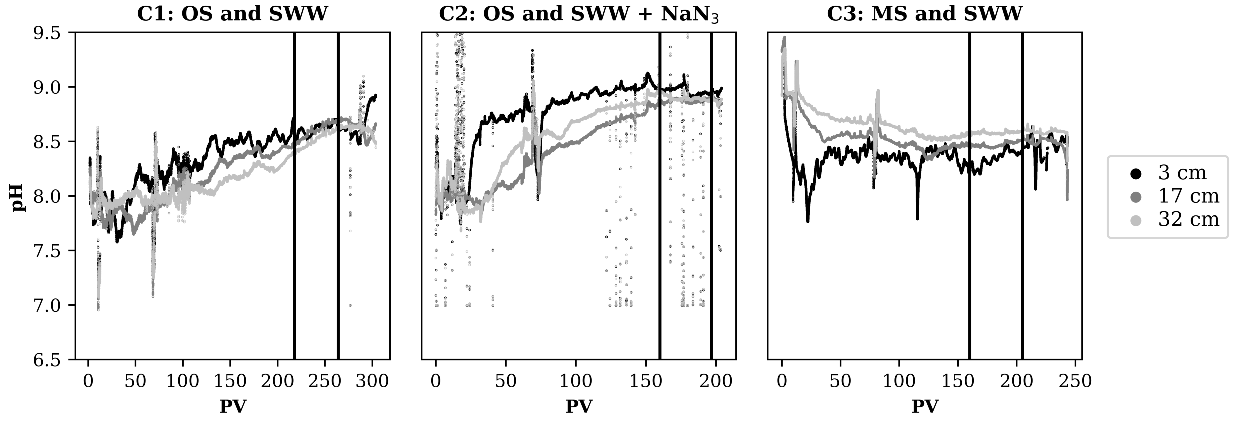

Figure S11: Temporal evolution of soil pH in the column experiments. Black lines represent the period of the injection of PhACs for each column.

The point of zero charge (PZC) of each soil was measured by potentiometric titration according to Anda et al.[12] Equilibriums to pH < 4 were not performed to avoid calcite dissolution.

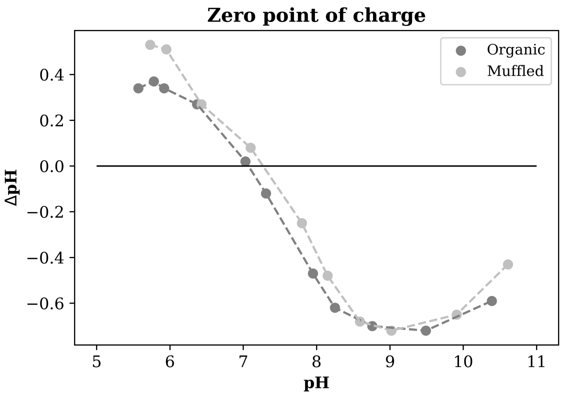

Figure S12: Point of zero charge determination of both soils.

S10 Temporal evolution of normalized hydraulic conductivity for the column experiments

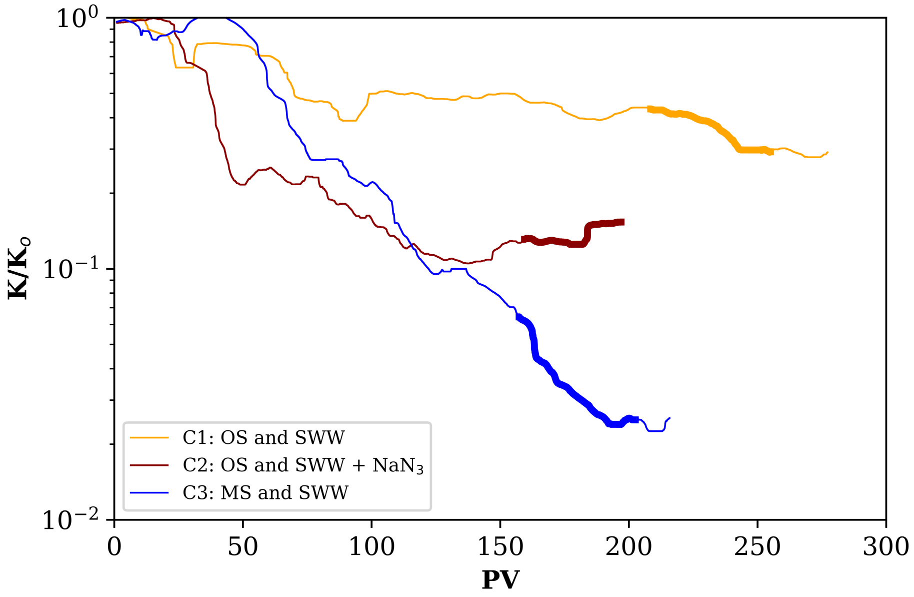

Figure S13: Temporal evolution of normalized hydraulic conductivity for the column experiments. K_o represents the initial hydraulic conductivity.

S11 Tracer results of column experiments

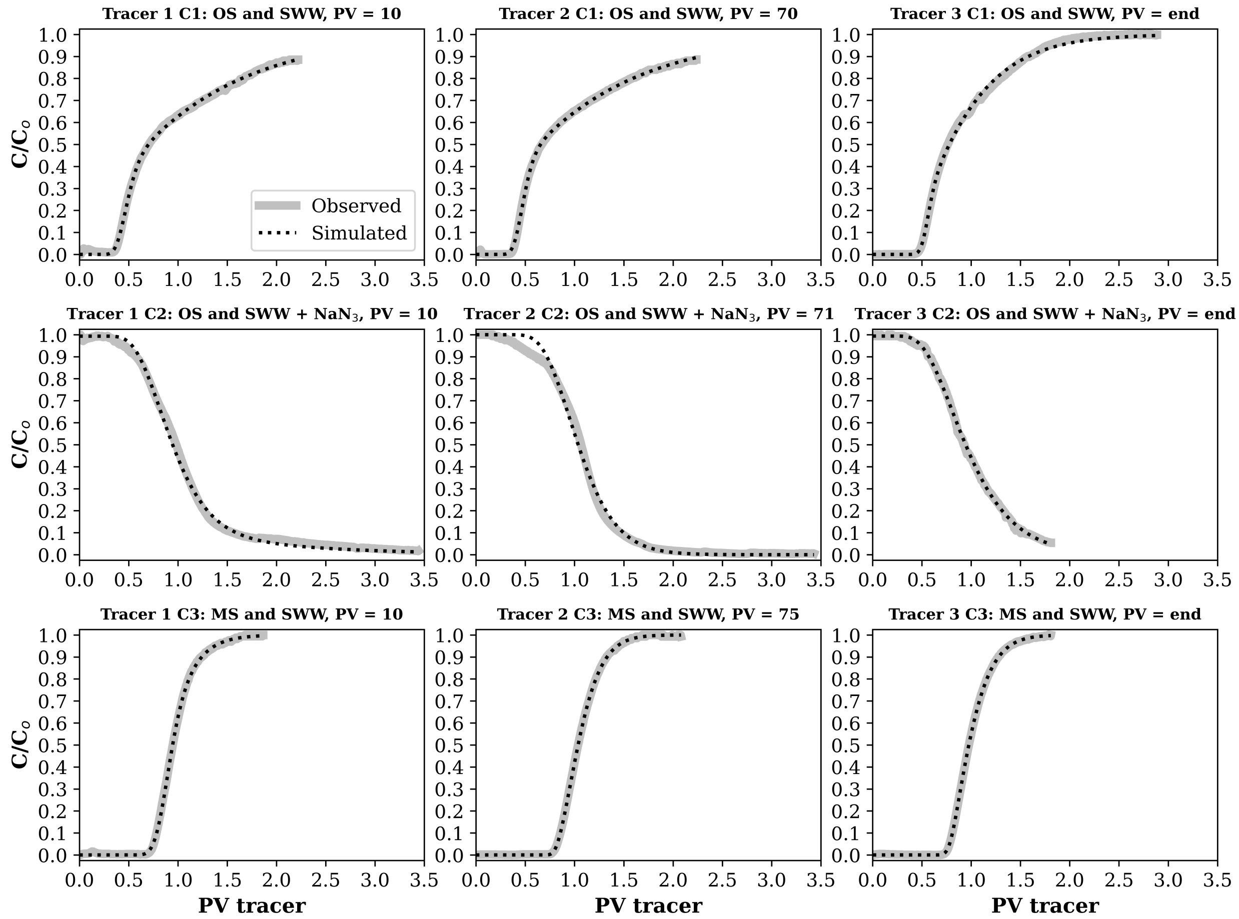

Figure S14: Observed and simulated results for the tracer experiments conducted in the columns.

S12 Protein and humic acid content of biofilms for column experiments

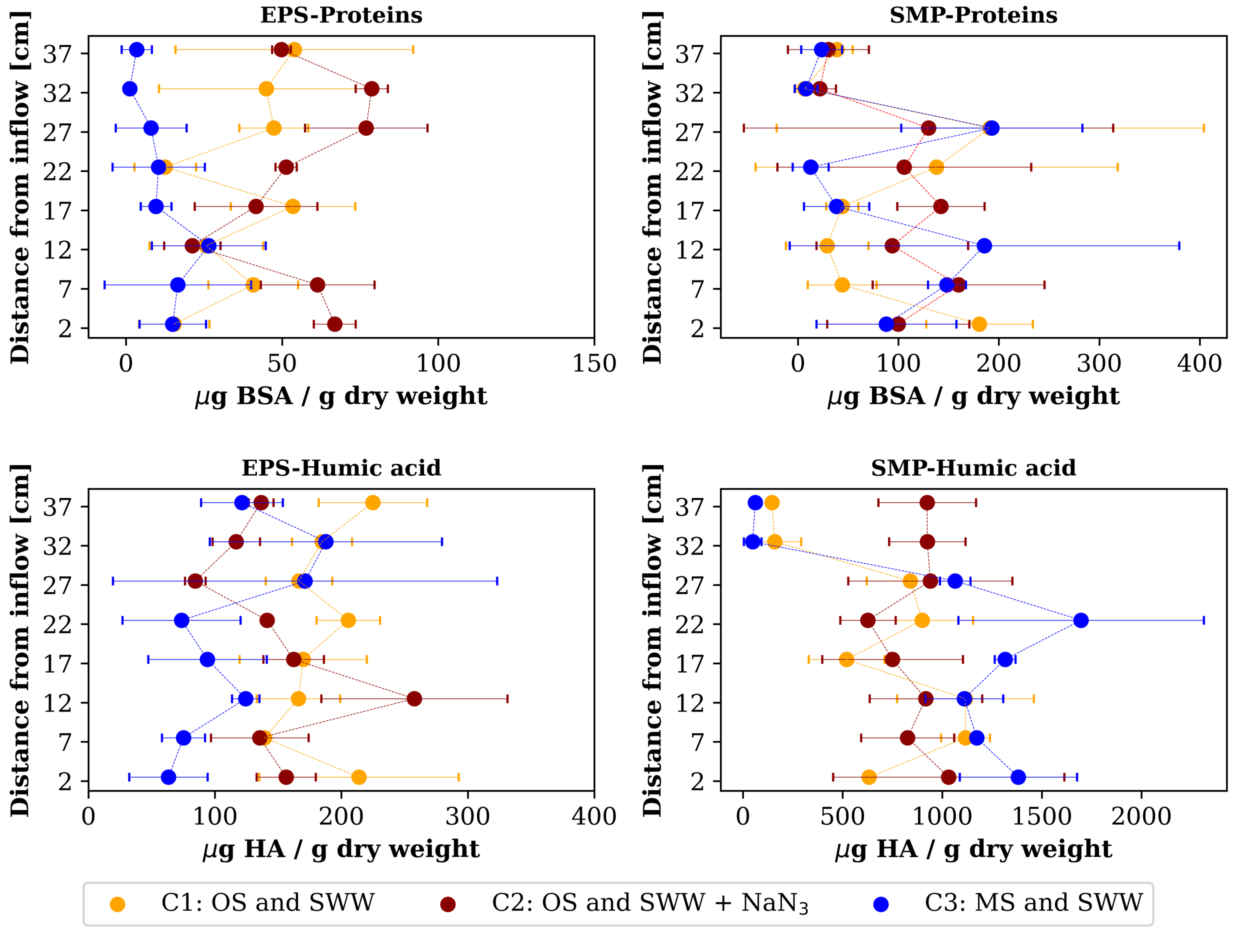

Figure S15: Protein and humic acid content of the biofilms at the end of column experiments. Error bars represent the standard deviation of triplicates.

No significant differences (p-value > 0.01) were found in the case of SMP-protein and EPS and SMP-humic acid content.

S13 Fitted parameters double porosity model for PhACs breakthrough curves in column experiments

Table S7: R and degradation rate obtained from breakthrough curves of column experiments. R is presented with a confidence interval of 95 %. μ is the degradation rate considering equal degradation rate in the liquid and solid phase.

Parameter	CBZ C1	CBZ C2	CBZ C3	DIC C1	DIC C2	DIC C3	MET C1	MET C2	MET C3
R	10.2	4.6	1.2	5.7	2.2	2.0	64.8	31.3	65.9
R_{min}	9.7	4.3	1.1	5.0	2.1	1.9	62.4	29.9	65.9
R_{max}	10.7	4.8	1.2	6.3	2.2	2.1	67.1	32.6	65.9
μ $[d^{-1}]$	0	0	0	$1.2 \cdot 10^{-2}$	$8.7 \cdot 10^{-3}$	0	$2.7 \cdot 10^{-2}$	$1.1 \cdot 10^{-2}$	0

S14 Results batch experiments

Table S8: Parameters of normalized Freundlich model for batch experiments.

**Soil**	**Parameter**	**CBZ**	**DIC**	**MET**
**Organic**	K^*_{Fr} [mg/kg]	$4.1 \cdot 10^1$	$3.0 \cdot 10^0$	$1.1 \cdot 10^4$
	n	1.09	0.66	1.00
	R^2	0.98	0.91	0.96
	p value	<0.01	<0.01	<0.01
**Muffled**	K^*_{Fr} [mg/kg]	-	-	$1.4 \cdot 10^4$
	n	-	-	0.72
	R^2	-	-	0.99
	p value	-	-	<0.01

Isotherms of CBZ and DIC in muffled soil were not determined because of no quantifiable sorption.

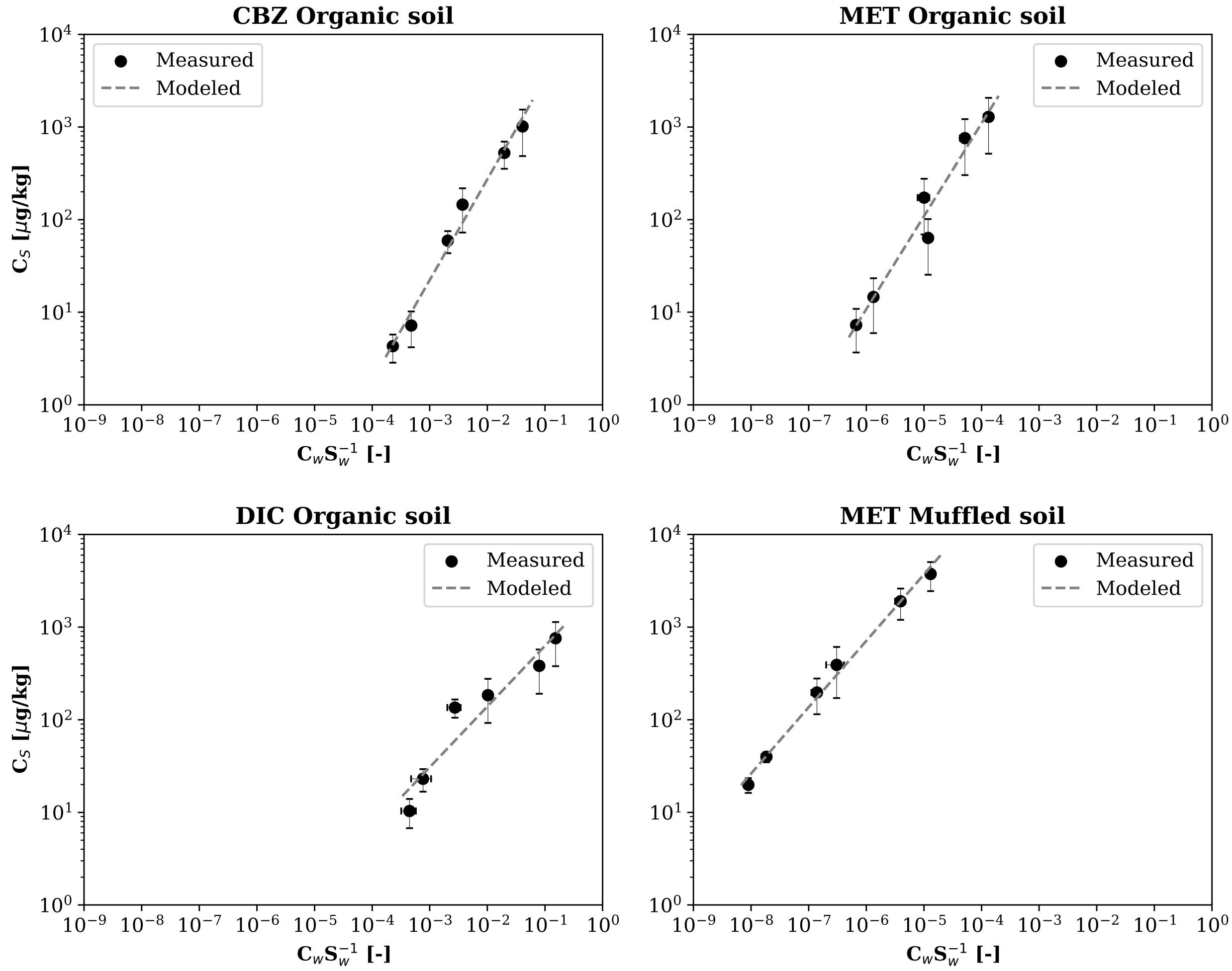

Figure S16: Freundlich normalized isotherms of batch experiments. Error bars correspond to standard deviation of triplicates.

References

(1) Jones, O.; Voulvoulis, N.; Lester, J. Aquatic environmental assessment of the top 25 English prescription pharmaceuticals. *Water Research* **2002**, *36*, 5013–5022.

(2) Schaffer, M.; Kröger, K. F.; Nödler, K.; Ayora, C.; Carrera, J.; Hernández, M.; Licha, T. Influence of a compost layer on the attenuation of 28 selected organic micropollutants under realistic soil aquifer treatment conditions: Insights from a large scale column experiment. *Water Research* **2015**, *74*, 110–121.

(3) Todeschini, R.; Vighi, M.; Finizio, A.; Gramatica, P. 30-Modelling and Prediction by WHIM Descriptors. Part 8. Toxicity and Physico-chemical Properties of Environmental Priority Chemicals by 2D-TI and 3D-WHIM Descriptors. *SAR and QSAR in Environmental Research* **1997**, *7*, 173–193.

(4) Li, J.; Wilkinson, J. L.; Boxall, A. B. Use of a large dataset to develop new models for estimating the sorption of active pharmaceutical ingredients in soils and sediments. *Journal of Hazardous Materials* **2021**, *415*, 125688.

(5) Chefetz, B.; Mualem, T.; Ben-Ari, J. Sorption and mobility of pharmaceutical compounds in soil irrigated with reclaimed wastewater. *Chemosphere* **2008**, *73*, 1335–1343.

(6) Niedbala, A.; Schaffer, M.; Licha, T.; Nödler, K.; Börnick, H.; Ruppert, H.; Worch, E. Influence of competing inorganic cations on the ion exchange equilibrium of the monovalent organic cation metoprolol on natural sediment. *Chemosphere* **2013**, *90*, 1945–1951.

(7) Bui, T. X.; Choi, H. Influence of ionic strength, anions, cations, and natural organic matter on the adsorption of pharmaceuticals to silica. *Chemosphere* **2010**, *80*, 681–686.

(8) *DIN 32645:2008-11, Chemische Analytik_- Nachweis-, Erfassungs- und Bestimmungsgrenze unter Wiederholbedingungen_- Begriffe, Verfahren, Auswertung*; Beuth Verlag GmbH, 2008.

(9) Redmile-Gordon, M.; Brookes, P.; Evershed, R.; Goulding, K.; Hirsch, P. Measuring the soil-microbial interface: Extraction of extracellular polymeric substances (EPS) from soil biofilms. *Soil Biology and Biochemistry* **2014**, *72*, 163–171.

(10) Redmile-Gordon, M.; Armenise, E.; White, R.; Hirsch, P.; Goulding, K. A comparison of two colorimetric assays, based upon Lowry and Bradford techniques, to estimate total protein in soil extracts. *Soil Biology and Biochemistry* **2013**, *67*, 166–173.

(11) Dreywood, R. Qualitative Test for Carbohydrate Material. *Industrial & Engineering Chemistry Analytical Edition* **1946**, *18*, 499–499.

(12) Anda, M.; Shamshuddin, J.; Fauziah, C.; Omar, S. S. Mineralogy and factors controlling charge development of three Oxisols developed from different parent materials. *Geoderma* **2008**, *143*, 153–167.
